# Fungal communities associated with early immature tubers of wild *Gastrodia elata*

**DOI:** 10.1002/ece3.11004

**Published:** 2024-02-21

**Authors:** Dong Li, Xiao‐Han Jin, Yu Li, Yu‐Chuan Wang, Hai‐Yan He, Han‐Bo Zhang

**Affiliations:** ^1^ State Key Laboratory Conservation and Utilization of Bio‐Resources in Yunnan Kunming China; ^2^ School of Ecology and Environmental Science Yunnan University Kunming China; ^3^ Gastrodia Tuber Research Institute of Zhaotong Zhaotong China; ^4^ The Agriculture and Life Sciences College Zhaotong University Zhaotong China; ^5^ Yunnan Key Laboratory of Gastrodia elata and Fungus Symbiotic Biology Zhaotong China

**Keywords:** DNA barcoding, growth‐promoting fungi, *Mycena plumipes*, next‐generation sequencing, nonphotosynthetic, Orchidaceae, targeted amplicon sequencing

## Abstract

Full myco‐heterotrophic orchid *Gastrodia elata* Bl. is widely distributed in Northeast Asia, and previous research has not fully investigated the symbiotic fungal community of its early immature tubers. This study utilized Illumina sequencing to compare symbiotic fungal communities in natural *G. elata* immature tubers and their habitats. LEfSe (Linear Discriminant Analysis Effect Size) was used to screen for Biomarkers that could explain variations among different fungal communities, and correlation analyses were performed among Biomarkers and other common orchid mycorrhizal fungi. Our results illustrate that the symbiotic fungal communities of immature *G. elata* tubers cannot be simply interpreted as subsets of the environmental fungal communities because some key members cannot be traced back to the environment. The early growth of *G. elata* was related to a small group of fungi, such as *Sebacina*, *Thelephora*, and *Inocybe*, which were also common mycorrhizal fungi from other orchids. In addition, *Mycena*, *Auricularia*, and *Cryptococcus* were unique fungal partners of *G. elata*, and many new species have yet to be discovered. Possible symbiotic *Mycena* should be *M. plumipes* and its sibling species in this case. Our results provide insight into the symbiotic partner switch and trophic pattern change during the development and maturation of *G. elata*.

## Graphical abstract

Full myco‐heterotrophic orchid *Gastrodia elata* Bl. is widely distributed in Northeast Asia, and previous research has not fully investigated the symbiotic fungal community of its early immature tubers. Illumina sequencing and a series of mainstream statistical analyses were utilized to compare symbiotic fungal communities in natural *G. elata* immature tubers and their habitats. Our results illustrate that the symbiotic fungal communities of immature *G. elata* tubers cannot be simply interpreted as subsets of the environmental fungal communities and the early growth was related to a small group of fungi, such as Sebacina, Thelephora, and Inocybe, which were also common mycorrhizal fungi from other orchids. *Mycena*, *Auricularia*, and *Cryptococcus* were unique fungal partners of *G. elata*, and many new species have yet to be discovered. Possible symbiotic *Mycena* should be *M. plumipes* and its sibling species.

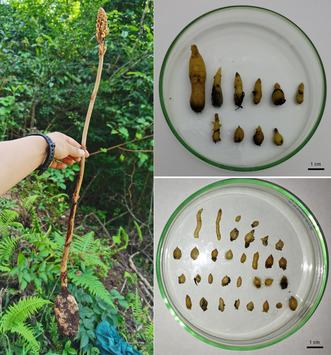

## 1 INTRODUCTION

Orchidaceae is one of the most diverse and widely distributed plant families on earth (Merckx, 2013). They were found in every habitat in the biosphere except glaciers and deserts, while most species were found in tropical and subtropical regions (Liu, Chen, et al., 2015). There were approximately 26,000 known species of Orchidaceae, distributed in nearly 800 genera (Govaerts, 2022). Like many other wild plants, orchids are also facing unprecedented threats from habitat fragmentation and destruction, excessive collection, climate change, and a range of other human‐caused problems (Phillips et al., 2020). Orchids have many irreplaceable values in the food industry and traditional medicine. *Vanilla planifolia* and its extractive Vanillin have been widely used in cosmetics and food industries (Blank et al., 1992) and *Dendrobium candidum* has always been a prized herb in traditional Chinese medicine (Ng et al., 2012). Ecologically, orchids are also very important. Orchids are often viewed as indicators of overall environmental health, and their special trophic modes can accelerate the flow of energy and material in the ecosystem while fixing inorganic carbon and energy (The Smithsonian Environmental Research Center, 2022). Finally, these highly variable metabolites of orchids make them inestimable in potential value, while it is also ethically and morally unacceptable to let such an important part of nature disappear, thus orchids are often considered a flagship taxon for global plant conservations (Liu, Chen, et al., 2015).

The seeds of orchids are tiny and nutrient‐deficient, relying on symbiotic fungi to nourish their germination. This trait was known as Initial Myco‐heterotrophy (Leake, 1994; Rasmussen & Rasmussen, 2009; Stockel et al., 2014). Seedlings of most orchids are known as Partial Myco‐heterotrophic or Facultative Myco‐heterotrophic because they can produce chlorophyll for photosynthesis after certain stages of growth. The adult plants of partial myco‐heterotrophic orchids were commonly assumed to be autotrophic, despite their utilization of varying amounts of carbon from fungi (Hynson et al., 2013). However, more than 200 orchid species had been found unable to photosynthesize and completely dependent on nutrients from their fungal symbionts throughout their whole life histories, called Full Myco‐heterotrophic or Obligate Myco‐heterotrophic orchids (Leake, 1994). The symbiotic fungi of orchids can be roughly divided into three groups: rhizoctonioid, mycorrhizal fungi, and saprophytic non‐rhizoctonioid and most of the orchid symbiotic fungi were confirmed to be Basidiomycetes, while Ascomycetes only occupied a very small part in previous researches (Dearnaley et al., 2012). Compared with facultative ones, full myco‐heterotrophic orchids were more deemed to be associated with saprotrophic non‐rhizoctonioid as they were less likely to provide any photosynthetic carbon to their fungal partners, although more recent studies have also detected rhizoctonioid and mycorrhizal fungi from them (Favre‐Godal et al., 2020; Kinoshita et al., 2016). Due to the unique trophic patterns of orchids, the environmental presence and distribution of symbiotic fungi were considered to be important influencing factors for the presence and distribution of endangered orchids (Phillips et al., 2020). It is undoubtedly of great significance in ecology theory and practical application to explore the structure and distribution of symbiotic fungal communities in orchids.

*Gastrodia* R.Br. is one of the largest full myco‐heterotrophic orchid genera, consisting of ca. 100 accepted species, distributed in eastern and southern Asia, Australasia, and southern Africa (POWO, 2023). The species composition and community structure of the fungal symbionts of Gastrodia, as well as whether they would vary with different host species, are all questions that require further investigation (Lee et al., 2015). The fungal partners of *Gastrodia* are generally considered to include Mycenaceae, Marasmiaceae, Russulaceae, Polyporaceae, Meruliaceae, Sebacinaceae, and Ceratobasidiaceae (Kinoshita et al., 2016; Lee et al., 2015; Li et al., 2022). *Gastrodia elata* Bl. is one of the representative species and is famous for its time‐tested applications in traditional Chinese medicine. Modern medicine has also archived the value of *G. elata* extracts such as Gastrodin and 4‐hydroxybenzaldehyde in treating diseases such as Brain Ischemia, Neurasthenia, and Depression (Chen & Sheen, 2011). Although related cultivation technology and practice have achieved certain achievements, natural *G. elata* populations are still facing threats and are rated Vulnerable on the IUCN Red List (IUCN, 2022). As an important orchid, the symbiotic fungi of *G. elata* was not yet clear. In Chinese academia, the most acknowledged researches on symbiotic fungi of *G. elata* were completed during the 1980s by Xu et al. (Xu & Guo, 2000). Based on classical culture‐dependent methods and morphological identification, they proposed that the mycorrhizal fungus at the early developmental stage of *G. elata* was *Mycena osmundicola*, which can facilitate seed germination and growth into protocorms. As *G. elata* tubers develop into the mature stage, the mycorrhizal fungus switches to *Armillaria mellea*. Although using *Mycena* for germination and *Armillaria* for maturation have already been commonly applied in the successful cultivation of *G. elata* in China (Hu et al., 2021), we could not find any strain or nucleic acid sequence evidence left by Xu et al. Most subsequent studies on the symbiotic fungi of *G. elata* were based on the accidental isolation of *Mycena* strains from the environment or other sources (Park & Lee, 2012), and *Mycena* isolations from *G. elata* tissues were rarely reported. On the other hand, common orchid mycorrhizal fungi usually belong to Tulasnellaceae, Ceratobasidiaceae, or Serendipitaceae instead of *Mycena* or *Armillaria* (Zhao et al., 2021). Excessive symbiotic specificity also contradicts the widely accepted view that Myco‐heterotrophy should be formed in the possible common ancestor of Orchidaceae (Ogura‐Tsujita et al., 2009). It is quite necessary to clarify the structure and distribution of symbiotic fungal communities associated with wild *G. elata* populations.

Classical methods of isolation and culture have been proven to be insufficiently effective because they have lost sight of over 99.99% of microorganisms in the environment (Bodor et al., 2020). The emergence and development of DNA barcoding methods and second‐generation sequencing technologies such as Illumina® sequencing have greatly expanded our awareness of these microorganisms that live inside, outside, or on the surface of a random plant. These technologies have also been proven to be effective in exploring symbiotic fungal communities associated with a variety of genera from Orchidaceae (Jacquemyn et al., 2014) including *Gastrodia* (Liu, Li, et al., 2015). We believe that the culture‐independent method can greatly improve our knowledge about *G. elata* fungal symbionts. Therefore, in our previous research, we investigated the symbiotic and environmental fungal communities of domestic *G. elata* tubers from one of their provenances by Illunina® sequencing (Chen et al., 2019). The results not only validated the feasibility of our approach but also revealed more complex fungal communities that included basidiomycetes and ascomycetes than expected and their variation trend with host growth and development.

To further demonstrate the symbiotic and environmental fungal communities of immature *G. elata* tubers in the wild, in this research, we collected samples of immature *G. elata* tubers and their growing environments from larger scale provenances. We expected to provide updated evidence of *G. elata* symbionts, point out the keystone fungal taxa, and put forward our views on the related scientific issues. In this article, we were trying to answer the following questions: (1) Do immature *G. elata* tubers have more symbiotic fungi besides the traditionally believed *M. osmundicola* or *A. mellea*? (2) What are the potential interactions and functions of these symbiotic fungi? (3) What are the environmental sources and structural characteristics of symbiotic fungal communities of immature *G. elata* tubers? and (4) Are there specificities and species preferences in their species recruitment?

## 2 MATERIALS AND METHODS

### 2.1 Research sites and sampling

Zhaotong City, Yunnan Province, PRC is the most respected *G. elata* provenance in traditional Chinese medicine. By visiting local people and investigating natural *G. elata* resources, the research sites were selected in four areas with relatively abundant natural *G. elata* resources within the jurisdiction of Zhaotong City: Panhe Town, Zhaoyang District (27°30′2″ N, 103°49′46″ E, 2029 m asl), Haizi Town, Yiliang County (27°30′25″ N, 104°18′2″ E, 2049 m asl), Manbu Town, Zhenxiong County (27°33′46″ N, 104°49′50″ E, 1759 m asl), and Lianfeng Town, Yongshan County (27°54′28″ N, 103°38′58″ E, 2167 m asl, Figure S1 & S2). Site Panhe was under a mixed broadleaf‐conifer forest composed mainly of unidentified pines and oaks with a herbaceous layer dominated by *Parathelypteris*. The litter was dominated by the remains of these plants, covering loose sandy soil. Site Haizi had a similar formation to Site Lianfeng: broad‐leaved Fagaceae trees dominated the tree layer, while broad‐leaved plants dominated the herb layer and the shrub layer. The differences lie in the scattered distribution of bamboo above finer and more uniform soil at Site Haizi but no bamboo was found at Site Lianfeng while the soil was mixing more gravel with a diameter of approximately 1 cm. Site Mangbu had an obviously different formation from the other three sites. The tree layer was almost entirely composed of some unidentified cypresses, with only a sporadic distribution of shrubs and almost no observed herbaceous plants. It also had the richest forest litter layer and the finest uniformly sticky soil.

Sampling took place between June 25 and 28, 2020. With the assistance of local guides, small amounts of wild *G. elata* tubers were found and collected with soil. The forest litter and soil were collected separately as environmental backgrounds in the area of approximately 50 m^2^ where there was no wild *G. elata* growing. At least three replicates were collected for forest litter and soil samples at each site and all samples were stored in portable cryogenic boxes and brought back to the laboratory. The number of sample replicate was set according to the “Rule of 10” by Gotelli & Ellison (2004). Considering the possible higher variability in *G. elata* symbiotic community, the number of replicate was appropriately increased, therefore, appropriate biological replicates and academic ethics requirements were well considered during the sampling process. Sampled tubers were carefully identified and separated from the soil that enclosed them, removing as much of their surface attachment as possible, and the attached soil was collected and marked as rhizosphere soil. Meanwhile, the tubers were quickly surface sterilized (30 s submergence in 0.5% sodium hypochlorite, followed by three 30 s rinse steps in sterile distilled water. The water that completed the last rinse step was sampled and spread on potato dextrose agar medium plates to verify the disinfection effect). All samples including *G. elata* tubers (Group Y), rhizosphere soils (Group YT), background litters (Group IY), and background soils (Group IT) were promptly mailed to LC‐Bio Technology Co., Ltd (Hangzhou, Zhejiang Province, PRC) with dry ice for subsequent amplicon sequencing with a few exceptions kept in the −80°C refrigerator of our laboratory.

### 2.2 Molecular analyses

Different types of samples were pretreated by different methods into smaller, more uniform fragments and then homogenized in liquid nitrogen. DNA was extracted using the classical CTAB (Cetyltrimethylammonium Bromide) method, and the extraction reagents were prepared by our technical service provider. The extracted DNA was inspected by agarose gel electrophoresis and quantified by UV spectrophotometry, then stored at −80°C and used as templates for subsequent PCR amplifications.

Internal transcribed spacer (ITS) regions of the ribosomal DNA were chosen as PCR amplification targets for fungal DNA barcoding (Schoch et al., 2012). The forward primer fITS7 (5′‐GTGARTCATCGAATCTTTG‐3′) and the reverse primer ITS4 (5′‐TCCTCCGCTTATTGATATGC‐3′) (Karlsson et al., 2014) were selected to amplify the targeted ITS2 region. The premix reagent used for the PCR reaction was Pusion Hot start flex 2× Master Mix (NEB, Singapore). The gradient thermal cycler used was LongGene A200 (LongGene, Hangzhou, PRC). PCR amplification was performed in a total volume of 25 μL reaction mixture containing 25 ng of template DNA, 12.5 μL PCR Premix, and 2.5 μL of each primer. The PCR conditions to amplify the ITS fragments consisted of an initial denaturation at 98°C for 30 s; 32 cycles of denaturation at 98°C for 10 s, annealing at 54°C for 30 s, and extension at 72°C for 45 s; and then final extension at 72°C for 10 min. The PCR products were confirmed by 2% agarose gel electrophoresis, then purified by AMPure XT beads (Beckman Coulter Genomics, Danvers, MA, USA) and quantified by Qubit (Invitrogen, USA).

The amplicon pools were prepared for sequencing and the size and quantity of the amplicon library were assessed on an Agilent 2100 Bioanalyzer (Agilent, USA) and with the Library Quantification Kit for Illumina® (Kapa Biosciences, Woburn, MA, USA), respectively. The libraries were sequenced on a NovaSeq 6000 platform (Illumina®, San Diego, CA, USA).

These technical details of PCR amplification and sequencing above were compiled according to the guidance document of our technical service provider.

### 2.3 Bioinformatics and statistical analysis

Sequencing data were deposited in the Sequence Read Archive (Sayers et al., 2022) (http://www.ncbi.nlm.nih.gov/sra). Clean data were obtained after double‐end splicing by overlap, quality control, and chimera filtering. Divisive Amplicon Denoising Algorithm (DADA2) (Callahan et al., 2016) of the QIIME 2 platform (Bolyen et al., 2019) was used to dereplicate, denoise, and form Amplicon Sequence Variant (ASV) (Blaxter et al., 2005) feature tables. Next, the characteristics of fungal communities among different sample groups were compared. Common and unique ASVs, alpha and beta diversity, etc., were calculated and displayed using R packages and QIIME2 processes. Taxonomic annotation was performed by the QIIME2 plugin feature‐classifier, and the alignment databases were RDP (Cole et al., 2014) and UNITE (Nilsson et al., 2019).

To better characterize the differences among sample groups, emphasizing both statistical significance and biological relevance, linear discriminant analysis (LDA) effect size (LEfSe) (Segata et al., 2011) was applied to the ASV feature tables after taxonomic annotation. ASV feature tables of different taxonomic levels were restructured according to the user guidance of the Galaxy platform (Afgan et al., 2018). The threshold on the logarithmic LDA score for discriminative features was set to 4.0 or 3.5 and the strategy for multiclass analysis was set as “one‐against‐all (less strict)” while other parameters remained the default. Before the cladogram was plotted, the results of LEfSe had been properly simplified according to the method proposed by Zhao and Liu (2019), low significant branches were hidden so biomarkers were better highlighted.

Based on the needs of production practices, more attention should be paid to the fungal taxa that have the potential to promote the growth of *G. elata* tubers. On the contrary, it is extremely difficult to isolate and purify all fungal species detected in *G. elata* tubers for functional verification in a short period of time. To appropriately simplify the research question, based on the fungal communities we detected in sampled tubers (group Y), combined with the results of genus‐level abundance rankings, representatives of community differences among groups (Biomarkers), and recommendations from previous research, a possible range was delineated for fungal ASVs that might promote the growth of *G. elata* tubers (PGPFASVs). The possible environmental origins of PGPFASV were then explored, and to further explore the possible correlation between these PGPFASVs, correlation analyses based on the Spearman method were conducted. To compare the similarities and differences in the environmental sources among PGPF genera, cluster analyses were conducted.

To better understand the evolutionary relationships between these unclassified but interesting PGPFASVs and their known sibling fungi, we searched and collected several ITS sequences of sibling fungi from the National Centre for Biotechnology Information (NCBI) Nucleotide database (Sayers et al., 2022) (https://www.ncbi.nlm.nih.gov/nuccore), ensuring that most of them were from type materials, aligned them with our biomarker sequences and constructed phylogenetic trees using MEGA 11 software (Tamura et al., 2021). We submitted Biomarkers sequences of interest in bulk to NCBI's online version of the Basic Loacal Alignment Search Tool (BLASTn) (https://blast.ncbi.nlm.nih.gov/Blast.cgi?PROGRAM=blastn&PAGE_TYPE=BlastSearch&LINK_LOC=blasthome) (Zhang et al., 2000) and searched rRNA/ITS databases. The genus names were submitted and the option “Internal transcribed spacer region (ITS) from Fungi type and reference materials” was selected as targeted loci project information. In program selection, we optimized the process for highly similar sequences (megablast) while leaving other algorithm parameters as default. In the Alignment Hit Table of the search results, we press Per. Ident and Max Score rearranged each Biomarker's hits in descending order, leaving only the first 5 of each Biomarker's hits and removed the duplication within them. The method of multiple sequence alignment was selected as ClustalW (Thompson et al., 1994) with default parameters and the phylogenetic tree was inferred using the Neighbor‐Joining method (Saitou & Nei, 1987). The evolutionary distances were computed using the p‐distance method (Nei & Kumar, 2000) and in units of the number of base differences per site. All positions with less than 50% site coverage were eliminated, i.e., fewer than 50% alignment gaps, missing data, and ambiguous bases were allowed at any position (partial deletion option). Only the optimal trees were shown and the percentage of replicate trees in which the associated taxa clustered together in the bootstrap test (1000 replicates) were shown next to the branches (Felsenstein, 1985).

## 3 RESULTS

### 3.1 Illumina sequencing and ASV taxonomic annotation

The tubers of *G. elata* that we sampled were small and in the early stage of their life cycle (Figure S3). In total, we sequenced 66 samples including 28 samples of natural *G. elata* tubers (Group Y), 11 samples of rhizosphere soils (Group YT), 15 samples of background litter (Group IY), and 12 samples of background soils (Group IT). Our sequencing depths were sufficient and low‐abundant ASVs could be well covered (Figure S4). The clean sequence read data of this research was deposited in the Sequence Read Archive under BioProject ID PRJNA962295. DADA2 dereplicate and denoise process converted all reads into 14,128 fungal ASVs. After taxonomic annotation, these fungal ASVs could be divided into 14 phyla, 60 classes, 146 orders, 396 families, 847 genera, and 1567 species. If we only took Basidiomycota into consideration, there were 2577 Basidiomycetous ASVs that could be divided into 15 classes, 46 orders, 127 families, 242 genera, and 520 species.

### 3.2 Comparison of fungal communities among different sample groups

**FIGURE 1:**
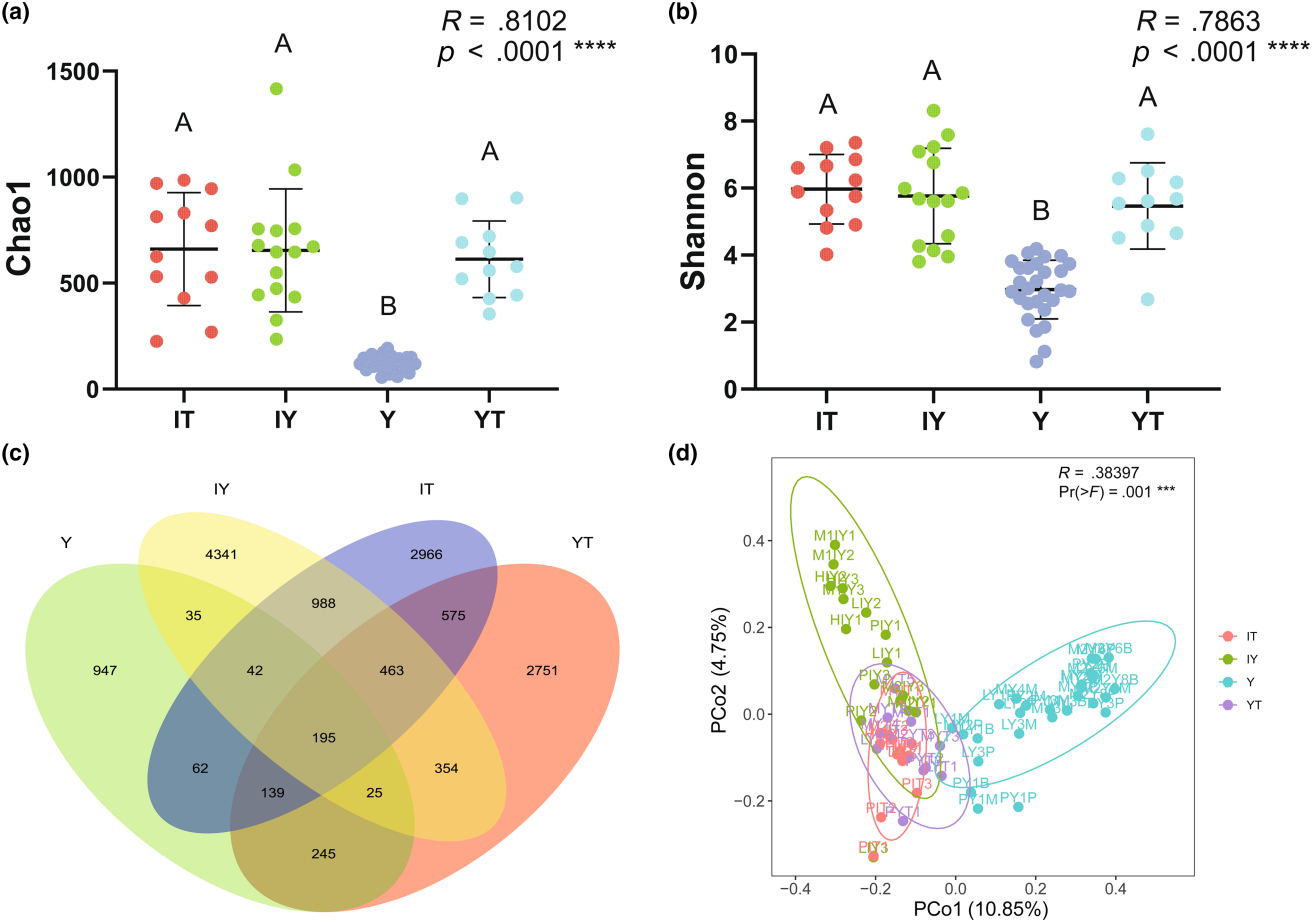
Comparison of basic information of fungal communities among four sample groups. (a, b) Comparison of alpha diversity based on Chao1 and Shannon–Wienner indices. There were significant differences between groups indicated by capital letters A, B. (c) Venn diagram illustrating the numbers of common and unique fungal ASVs among sample groups. (d) Results of beta diversity comparison demonstrated by PCoA and Adonis. Specific calculated results were shown in the Supporting Information. IT, background soils; IY, background litters; Y, *Gastrodia elata* tubers; YT, rhizosphere soils.

Common and unique ASVs among different sample groups are illustrated in Figure 1c. In total, the fungal communities from *G. elata* tubers (group Y) could be characterized by 1690 ASVs and were the fewest, of which 56.03% were unique. The fungal communities from rhizosphere soils (group YT), background soils (group IT), and litters (group IY) contained many more ASVs than group Y. Group Y shared 35.74%, 25.92%, and 17.57% of its fungal ASVs with group YT, group IT, and group Y, respectively. Alpha diversity of group Y was significantly lower while there was no significant difference between the other three groups (Figure 1a,b, Table S1 & S2). Beta diversity comparison results showed that sample group Y could be well distinguished from group IY and group IT and retained only a small overlap with group YT (Figure 1d). Adonis results further showed significant differences among groups (Table S3).

**FIGURE 2:**
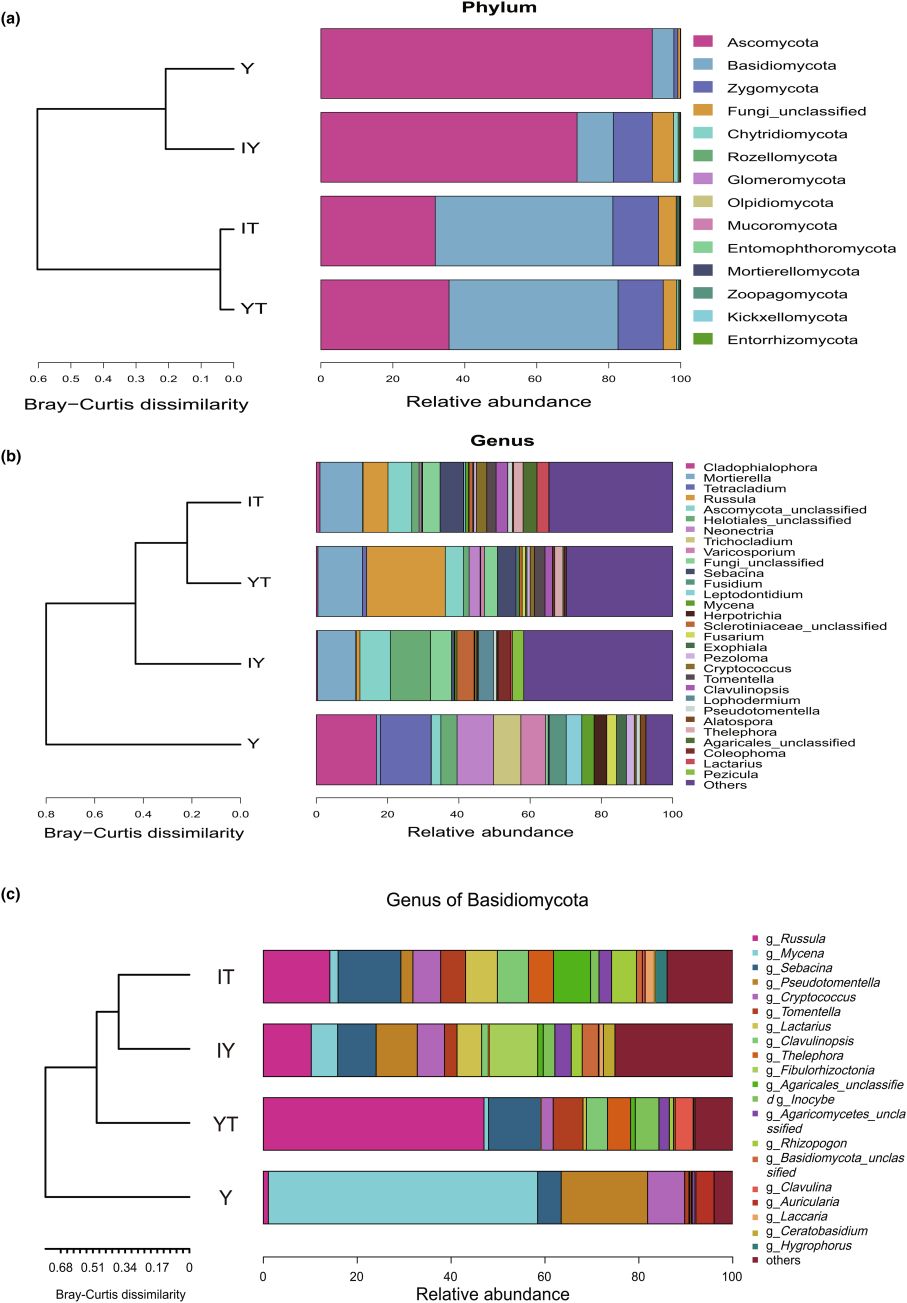
Staked bar charts illustrating the relative abundances of different sample groups at different taxonomic levels and community ranges. (a) At phylum level; (b) all fungal phyla at genus level; (c) only Basidiomycota at genus level. The cladograms on the left showed the results of clustering analysis among sample groups. IT, background soils; IY, background litters; Y, *Gastrodia elata* tubers; YT, rhizosphere soils.

The clustering analysis at different taxonomic levels among sample groups (Figure 2) showed more details about intergroup differences. At both the phylum and genus levels (Figure 2a,b), group Y was similar to group IY. However, when only considering the genera from Basidiomycota (Figure 2c), group Y became closer to group YT. For *G. elata* tubers (group Y), Ascomycetes occupied most of the fungal community, with the top 5 most abundant genera being *Cladophialophora*, *Tetracladium*, *Neonectria*, *Trichocladium* and a group of unclassified fungi. However, if we only took Basidiomycota into consideration, the top 10 genera were *Mycena*, *Pseudotomentella*, *Cryptococcus*, *Sebacina*, *Auricularia*, *Scopuloides*, *Russula*, *Tomentella*, a genus of unclassified Agaricomycetes and *Ramariopsis*. Interestingly, the proportions of *Russula* were quite high in the three background groups, especially in group YT, but a very low proportion in group Y.

**FIGURE 3:**
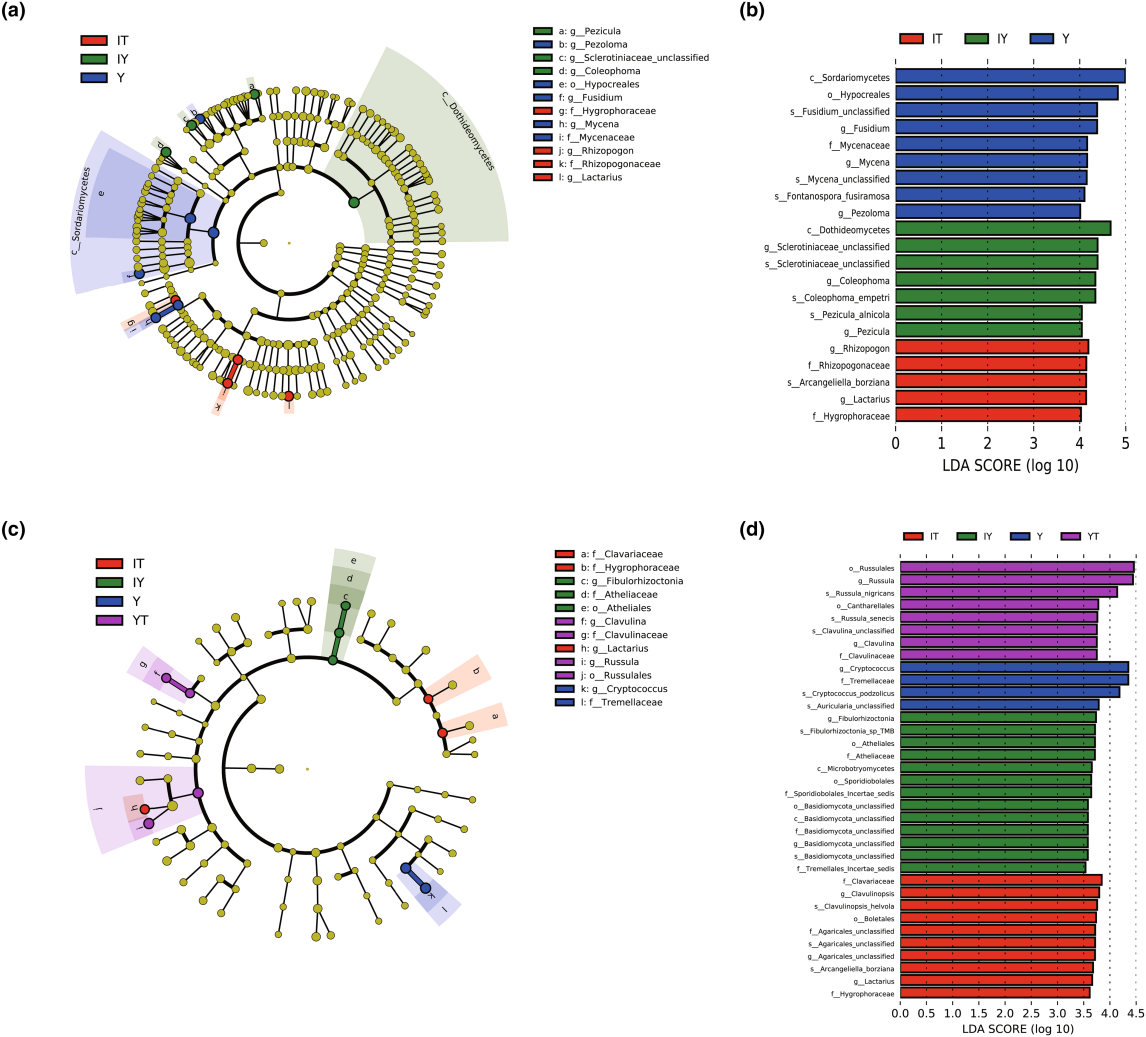
LEfSe results illustrating the different levels of taxa with significant relative abundance changes (a.k.a. biomarkers) among different sample groups. (a, b) Considering all phyla of fungi; (c, d) only considering Basidiomycota. (a, c) Cladogram illustrating the evolutionary relationships among biomarkers; (b, d) biomarkers with LDA scores greater than 4.0 (3.5 for d). IT, background soils; IY, background litters; Y, *Gastrodia elata* tubers; YT, rhizosphere soils.

LEfSe results showed a total of 21 biomarkers with LDA scores greater than 4.0 were screened out (Figure 3a,b). In total, 21 discriminative biomarkers could be recombined into two phyla, five classes, seven orders, nine families, nine genera, and seven species (details can be found in Tables S4–S6). In total, nine biomarkers of group Y could be recombined into two phyla, three classes, four orders, three families, four genera, and three species. For their taxonomic relationships, three relatively complete branches of the phylogenetic tree could be traced back from *Fontanospora fusiramosa*, some unclassified species of *Mycena* (Figure 3a(i)) and *Fusidium* (Figure 3a(f)), while two incomplete short branches terminated at Hypocreales (Figure 3a(e)) and *Pezoloma* (Figure 3a(b)). Again, if we only took Basidiomycota into consideration and reduced the threshold of LDA score to 3.5, 35 discriminative biomarkers could be recombined into at least four classes, seven orders, eight families, seven genera, and eight species according to their taxonomic relationships (detail can be found in Tables S7–S10). For four biomarkers of group Y, two relatively complete branches of the phylogenetic tree could be traced back from *Cryptpcoccus podzolicus* (Figure 3c(k)) and some unclassified species of *Auricularia*.

### 3.3 Definition and traceability analysis of possible growth‐promoting fungi

**FIGURE 4:**
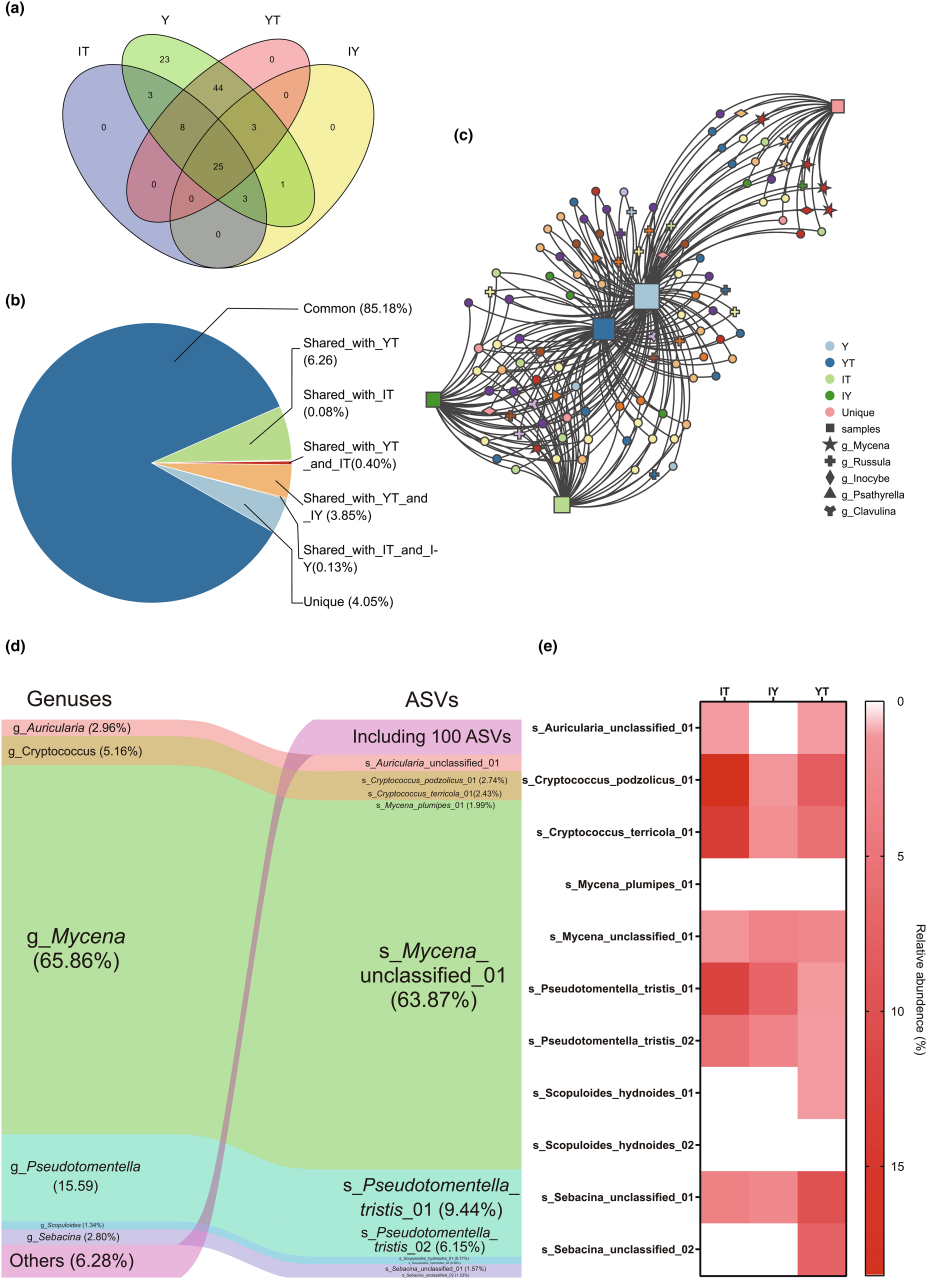
Source analysis of possible growth‐promoting fungi ASVs (PGPFASVs) in group Y. (a) Venn diagram illustrating the number of PGPFASVs shared by group Y with the other three groups. (b) Pie chart illustrating the source of PGPFASVs considering their relative abundance in group Y. (c) Net plot illustrating the source of PGPFASVs in group Y, among which different colors of nodes represent different sample groups and ASVs, and different shapes represent the taxonomic attribution of ASVs at the generic level. (d, e) Source analysis of PGPFASVs with relative abundance in the top 11 and higher than 0.01% in group Y. (d) Sankey diagrams showing composition and flow. (e) Heat map illustrates the presence of these 11 PGPFASVs in the other three groups.

A total of 110 ASVs belonging to 15 genera and at least 39 species were designated as possible growth‐promoting fungal ASVs (PGPFASVs) of *G. elata* (listed and appropriately renamed in Table S11). The Venn diagram (Figure 4a) and net plot (Figure 4c) illustrated that 23 ASVs (20.90% of the richness) were unique in group Y and could not be traced to the other three groups, including most *Mycena* ASVs (except only 1 that could be traced back to any of the other groups). In total, 44 ASVs (40.00% of the richness) could only be traced to group YT, 1 ASV (0.09% of the richness) to IY and 3 (2.73% of the richness) IT while some ASVs could be traced back to both two of the three groups and 25 ASVs (22.73% of the richness) were common among the four groups. The number of ASVs detected in different genera and their distribution in the environment varies. Fifteen genera that we designated as PGPF could be roughly divided into four categories (Figure S5). The first category contains only *Cryptococcus*. Twenty‐three out of the 110 PGPFASVs were from *Cryptococcus* and were the richest. These ASVs maintained higher variability and more unique ASVs were scattered in different sample groups. The second category contains *Psathytella*, *Clavulina*, *Thelephora*, *Ramariopsis*, and *Flagelloscypha*. Not a single PGPFASVs from these five genera was unique in group Y and confirmed to originate from the surrounding environments while all of these ASVs could be found in the rhizosphere soil (group YT). The third category contains *Russula*, *Sebacina*, and *Tomentella*. There were 15 or 16 PGPFASVs from these 3 genera respectively, and most of them could be traced back to the environment, especially the rhizosphere, leaving few exceptions that only existed in tubers. The last category contained *Mycena*, *Inocybe*, *Pseudotomentella*, *Tuber*, *Cryptococcus*, *Auricularia*, and *Scopuloides*. These genera contained fewer than 10 PGPFASVs and most of them were unique in tubers. If we further considered the read numbers and calculated the mean relative abundance of each ASV in group Y (Figure 4b), we would notice that common ASVs accounted for 85.18% of the total reads, while unique ASVs accounted for only 4.05%. The top 11 PGPFASVs (Table S12) were from *Mycena*, *Pseudotomentella*, *Cryptococcus*, *Auricularia*, *Sebacina*, and *Scopuloides*, which accounted for 93.72% of the total reads (Figure 4d). The top 1 PGPFASV was Mycena_unclassified_01, which occupied 63.87% of the total reads and was also the only *Mycena* ASV that could be traced back to the environment (Figure 4e). Most of the PGPFASVs ranked 2nd to 11th in abundance could also be traced back to the environment with only two exceptions: s_Mycena_plumipies_01 and s_Scopuloides_hydnoides_02. The second category that we previously divided by their origins was scarce in abundance. Genera and ASVs with high abundance were more likely to come from the fourth category which also contained the largest proportions of unique ASVs.

### 3.4 Correlation analyses in possible growth‐promoting fungal communities

**FIGURE 5:**
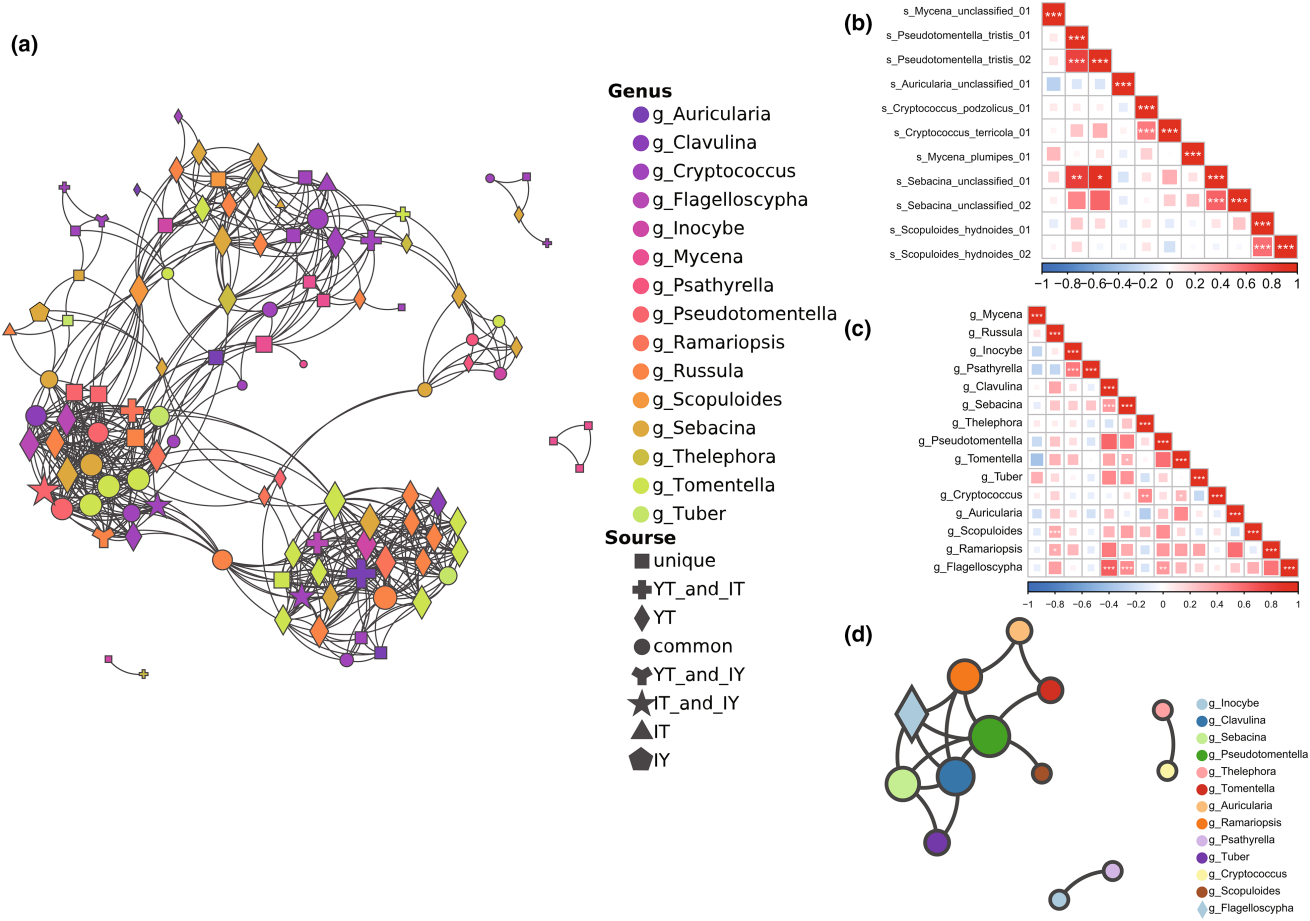
Correlation analysis of the 110 possible growth‐promoting ASVs in group Y. (a) Correlation net for all 110 ASVs; (b) correlation matrix for the top 11 ASV; (c, d) for all possible Orchid mycorrhizal fungi genuses. The correlation coefficient *R* was calculated by the Spearman method. “*” in figure a and c indicates the significance level of the correlation between the two, and only dominant links (with *R* > .4 or *p*‐value <.05) were shown in figure b and d.

If we ignored these differences among infraspecific ASVs and took all 15 genera of PGPFASVs into consideration, we could obtain the following results from the matrixed correlation heatmap (Figure 5c): the strongest and most significant correlations were between *Russula* and *Scopuloides*, *Inocybe* and *Psathyrella*, *Clavulina* and *Sebacina*, *Clavulina* and *Flagelloscypha*, *Sebacina*, and *Flagelloscypha*, *Thelephora* and *Cryptococcus*, *Pseudotomentella*, and *Flagelloscypha*. The net plot of dominant connections could be divided into three parts at this point, including one complex part centered on *Pseudotomentella*, *Flagelloscypha*, *Clavulina*, *Sebacina*, and *Ramariopsis*, and two simpler parts only showed branchless links between *Thelephora* and *Cryptococcus*, *Inocybe* and *Psathyrella* (Figure 5d). According to the correlation net at the genus level, there could be three relatively independent modules of interaction in these PGPF of *G. elata*. In each module, there were strong positive correlations between the confirmed orchid mycorrhizal fungi and other PGPFs.

### 3.5 Phylogenetic analysis of key fungal ASVs

**FIGURE 6:**
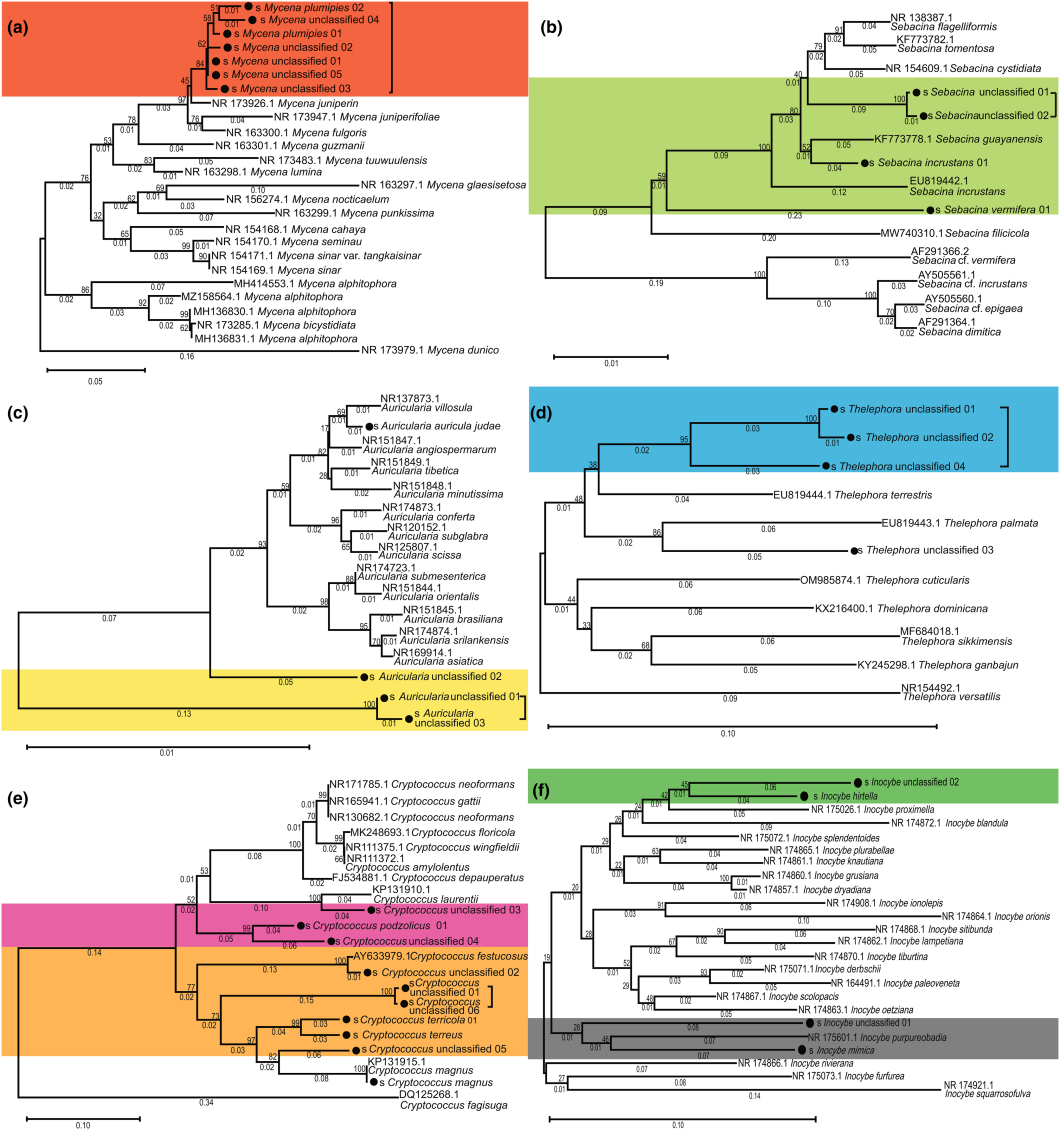
Phylogenetic trees illustrating the genetic relationships of unclassified *Mycena* (a), *Sebacina* (b), *Auricularia* (c), *Thelephora* (d), *Cryptococcus* (e), and *Inocybe* (f) ASVs from group Y to some known congeners. Black rounds marking the ASVs from this study.

Based on the three possible interaction modes mentioned above, six representative genera: *Mycena*, *Sebacina*, *Thelephora*, *Auricularia*, *Cryptococcus*, and *Inocybe* from PGPF were subjected to phylogenetic analysis (Figure 6). The results showed that the five unclassified *Mycena* ASVs had very close genetic distances from the two ASVs classified as *M. plumipes* and formed a relatively independent clade (Figure 6a). Four *Sebacina* ASVs were distributed in the phylogenetic tree, and two unclassified ASVs had a close genetic distance between them, while no relatively close known reference species were found (Figure 6b). Similarly, most ASVs belonging to *Auricularia*, *Thelephora*, *Cryptococcus*, and *Inocybe* formed a relatively independent clade that was genetically distant from known reference species (Figure 6c–f), suggesting that many PGPF from *G. elata* are potential novel species.

## 4 DISCUSSION

In this research, fungal communities associated with wild *G. elata* were investigated by Illumina® sequence technology. Non‐culture sequencing easily answered our first question and revealed a much more diverse symbiotic fungal community than traditionally believed. A total of 1690 ASVs from 239 possible genera were detected from the immature tubers of *G. elata* we sampled. The top 5 most abundant genera in immature tubers of *G. elata* were *Cladophialophora*, *Tetracladium*, *Neonectria*, *Trichocladium*, and a group of unclassified fungi. According to existing research, Basidiomycota received more attention and the top 10 basidiomycetous genera were *Mycena*, *Pseudotomentella*, *Cryptococcus*, *Sebacina*, *Auricularia*, *Scopuloides*, *Russula*, *Tomentella*, a genus of unclassified Agaricomycetes and *Ramariopsis*. Among them, *Mycena*, *Sebacina*, *Russula*, and *Tomentella* were suggested as orchid mycorrhizal fungi by Dearnaley et al. (2012), while other genera lacked evidence of orchid mycorrhizal formation. Since the possibilities of growth‐promoting effects of these non‐orchid mycorrhizal fungi cannot be ruled out, we focused on statistically demonstrating the significance of their variations across the environmental gradients and their correlations with known orchid mycorrhizal fungi when answering our second question about the potential interactions and functions of these symbiotic fungi.

Five biomarkers at the top of the clade in group Y have been shown to be more or less associated with orchids or mycorrhizas in known research. *Mycena* was first demonstrated as an early orchid mycorrhizal fungus of *G. elata* in the 1980s by Xu and Guo in Chinese academic circles and was later published worldwide (Xu & Guo, 2000). Although Hypocreales and Xylariales from Ascomycota were not on the orchid mycorrhizal fungi list of Dearnaley et al. (2012), a growing number of recent researches are proving the possibility of isolating or detecting them from orchids that grow in tropical areas of Madagascar (Yokoya et al., 2021), Sri Lanka (Ratnaweera et al., 2014), Thailand and southwestern China (Ma et al., 2022), Mexico (Avila‐Diaz et al., 2013; Beltrán‐Nambo et al., 2018) and Puerto Rico (Bayman et al., 1997). *Fontanospora fusiramosa* can be found in earlier evidence for endogeny in *Alnus* roots (Marvanová et al., 1997). *Pezoloma* was suggested to be an ericoid mycorrhizal fungus (Midgley et al., 2017). These evidence support the symbioses between Ascomycetes and *G. elata*. Based on these evidence, we speculated that the last two biomarkers, *F. fusiramosa* and *Pezoloma*, could indicated mycorrhizal cheating in *G. elata*—benefitting by tapping into the growth‐promoting network of neighbor plants, rather than forming its own (Bidartondo et al., 2004). However, stable isotope abundance evidence from congeneric *Gastrodia confusa* (Ogura‐Tsujita et al., 2009) and *Gastrodia sesamoides* (Dearnaley & Bougoure, 2010) showed significant differences between *Gastrodia* and ectomycorrhizal plants, which did not support our speculation. Since there was also ample evidence that the switching of symbiotic fungal partners occurred in different stages of growth and development for *G. elata* (Chen et al., 2019; Xu & Guo, 2000) but not for *G. confusa* or *G. sesamoides*, we further speculated that there could be some difference between the myco‐heterotrophic pattern of *G. elata* and the two congeneric species mentioned above. The selection pattern of symbiotic fungal partners may change dynamically with the life history of *G. elata* (Rasmussen et al., 2015: 391–402), and the nutrient supply capacity of symbiotic partners could also gradually increase. Changes in trophic patterns may have occurred during development and maturation, and more evidence from stable isotope abundances was expected to clarify whether this is mycorrhizal parasitism or a more general growth‐promoting effect. Morphological evidence did support our speculation of nutrient type differences since it is commonly believed that the more abundant and stable the source of nutrients is, the more likely for the plant to have a larger body size (James et al., 2012; Liu et al., 2010). The size and biomass of tubers and flower stems of *G. elata* during sexual reproduction (approximately 8–12 cm long, 5 cm diameter and 200 g for tubers, 200 cm tall for flower stems) (eFloras, 2022b; Xu & Guo, 2000) were higher than those of *G. confusa* (approximately 2–5 cm long for tubers and 15 cm tall for flower stems) (eFloras, 2022a) and *G. sesamoides* (approximately 8 cm long and 3 cm diameter for tubers, 12–75 cm tall for flower stems) (PlantNET, 2023). When we narrowed down the scope of fungi considered, 4 more biomarkers composed of 2 more branches were screened out. One of them was *Cryptococcus*, which was usually regarded as an endophyte or nectar‐contaminator in existing orchid microbiome researches (Jacquemyn et al., 2013; Xing et al., 2011). The other one was *Auricularia*, although less related to orchids, also had evidence of promoting seed germination of *Erythrorchis ochobiensis* (another achlorophyllous orchid) (Umata, 1997). Considering that similar growth‐promoting effects may also appear in *G. elata*, we temporarily considered it as a possible growth‐promoting fungus.

These results above not only further answered our first two questions but also triggered our thinking and speculation. Next, we were going to discuss our third and fourth questions, i.e., the environmental source and structural characteristics of the symbiotic community, combined with the species composition results of environmental fungal communities. The traditional view believed that the symbiotic fungi of *G. elata* should be acquired entirely from the surrounding environment, but our results revealed that there could be other source patterns. The symbiotic fungal communities were quite different forms and cannot be simply interpreted as subsets of the environmental ones since their differences in beta diversity and 56.03% of fungal ASV richness in group Y cannot be traced to the environment. Although we had narrowed down the range of fungi that we concerned into 15 genera, we could still observe 3 relatively different patterns of fungal origin at this time according to the clustering results (Figure S5). These different patterns not only reflect the characteristics of different microenvironments but also may imply the recruitment preference of *G. elata* for environmental fungi. Our previously published research demonstrated that there were indeed unexplained changes in the symbiotic fungal communities of *G. elata* tubers at different developmental stages (Chen et al., 2019). Combined with the results of this study, we further speculated that fungi that were relatively scarce in symbiotic communities and more dependent on environmental sources may have played their roles at earlier stages of germination and were gradually substituted by fungi that were more common in the environment. Correlation networks provided us with basic models of community structure and illustrated that these scarce PGPF genera tended to occupy more central positions in the network and held more correlative linkages. And these abundant PGPF genera located at the margin of the correlation network further proved the existence of preferences in species recruitment. For these abundant PGPF genera, the recruitment processes from the environment were more selective. For some of these genera, we successfully obtained biomarkers at the species level and even at the ASV level, suggesting these significant changes during recruitment were more concentrated in some specific genetic types.

Meanwhile considering the obvious differences among congeneric ASVs in the correlation analysis, it was quite necessary to conduct preliminary phylogenetic analysis of some important genera. Genetic distances calculated based on the ITS2 fragments were indeed not enough to prove whether those unclassified ASVs belong to some known or novel species, but it should have been enough to prove that they were not the former, thus we focused more on how these PGPFASVs differ from each other and from known species. Six representative genera: *Mycena*, *Sebacina*, *Thelephora*, *Auricularia*, *Cryptococcus*, and *Inocybe* from PGPF were subjected to phylogenetic analysis (Figure 6). For the abundant genus *Mycena* that occupied marginal nodes of the correlation net, the results showed that there were five unclassified *Mycena* ASVs that maintained close genetic distances from the two ASVs classified as *M. plumipes* and formed a relatively independent clade (Figure 6a). Since Xu and Guo first proposed *M. osmundicola* as the early symbiont of *G. elata* in the 1980s, relevant isolates and molecular biological evidence have been lacking. MK278400.1 was the only record in the NCBI nucleotide database about *M. osmundicola*, and its reliability is questionable. More recent systematic taxonomic researches suggest that *M. osmundicola* and *M. alphitophora* should be the same species (Na, 2019). In our results, there were long genetic distances between *M. alphitophora* records and our detected clade, which should be sufficient to confirm that neither *M. osmundicola* nor *M. alphitophora* was the early symbiote of *G. elata* in this case, and the possible symbiotic *Mycena* should be *M. plumipes* and its sibling species. This research could be the second piece of evidence since this rare *Mycena* species was first found and sequenced in Romania (Chinan & Fusu, 2016). Similar situations happened in the other 2 abundant but marginal genera including 3 unclassified *Auricularia* ASVs (Figure 6c) and 10 *Cryptococcus* ASVs (Figure 6e). *Sebacina*, *Thelephora*, and *Inocybe* were scarce but keystone genera of the correlation net. *Sebacina* and *Thelephora* used to be members of a group formerly known as “rhizoctonia” and are considered to be symbiotic fungi common among all orchid species (Zhao et al., 2021). Unclassified ASVs from all three genera maintained genetic diversification and tended to form independent branches without known siblings, indicating that they require more taxonomic researches (Figure 6b,d,f). Considering the differentiated diversities of these PGPF genera and their pending species classification, we believe that it is highly possible to discover novel species from them.

*Mycena* was indeed quite different from the other 14 PGPF genera. The environment did provide the most abundant *Mycena* ASV in the symbiotic fungal community, but not for the other six. Seventeen similar cases also happened in other PGPF genera. So, where did 23 of the 110 PGPFASVs that could not be traced to the environment come from? We believe that this is an issue worthy of further discussion. We speculated that the most likely source is parental vertical transmission through either sexual reproduction or asexual budding. There was evidence that orchids can transfer symbiotic fungi from other tissues to new roots (Calevo et al., 2021), thus it is also possible for *G. elata* to attach symbiotic fungi associated with germination and early growth during seed formation. On the contrary, due to the uncertainty of field sampling, it was possible indeed to contain young tubers that were produced by budding. Vertically transmitted fungi in both situations may not be able to survive and spread into the environment after the host tubers died, making it difficult to detect them in environmental samples (Calevo et al., 2021). Since there were also orchid nectar contaminators and mycorrhizal fungi of other plants detected in our tuber samples, we further hypothesized that these early symbiotic fungi may also be horizontally transmitted among different parents and even different species through pollination processes or mature mycorrhizal networks, but there are still few researches in these fields.

In addition, *Armillaria* was widely believed to be the dominant mycorrhizal partner in the late growth stage of *G. elata*. To our surprise, we did not detect any *Armillaria* ASV in our samples, even in the background environment, which makes us curious about the natural distribution of *Armillaria*. Generally, *Armillaria* was regarded as a virulent tree pathogen or saprophyte. Since the viability and infectivity of the basidia and haploid mycelia were usually low, *Armillaria* normally existed in soil and litter layers within the depth of approximately 30 cm in the form of large mycelia as rhizomorphs or mycelium mats, and infection occurs after direct contacted with plant roots (Devkota & Hammerschmidt, 2020; Przemieniecki et al., 2021). These large mycelia could have been removed during sample preprocessing prior to molecular analysis, leading to the absence of *Armillaria* in the three groups of environmental sample results of our research. On the other hand, the absence of Armillaria in group Y supported Xu and Guo's view (Xu & Guo, 2000) that *Armillaria* was not the symbiotic fungus at the early growth stage of *G. elata*, but we were skeptical of their view that the late symbiotic fungal species was *A. mellea* because it was generally considered to be highly lethal to many plants, especially trees (Devkota & Hammerschmidt, 2020). Stable symbiosis should be the prerequisite for the formation of high‐level myco‐heterotrophy (Suetsugu & Matsubayashi, 2021), and according to the classical ecological theory, stable symbiosis can be generated only when the negative impacts of the parasites on the hosts have become sufficiently low. *Armillaria* species that are most likely to form symbiosis and were potential intermediaries for mycorrhizal cheating according to our speculation, should not be so lethal and just maintain unstable relationships with adjacent photosynthetic plants since these plants were the important source producers of organic carbon. There were three aspects of evidence supporting our suspicion. The first aspect was that *Armillaria* species identification results based on classical methods were challenged, and many strains that were previously identified as *A. mellea* were later re‐identified as other *Armillaria* species. (Park et al., 2018). The second aspect was that not all *Armillaria* species were shown to be absolutely pathogenic to all common plants. The pathogenicity of *Armillaria* varies with specific species and hosts and is influenced by mycosphere microbes (Przemieniecki et al., 2021). Some *Armillaria* species were considered harmless (Przemieniecki et al., 2021) or not causing lethal diseases (Guo et al., 2016). *A. altimontana* was further shown to form a mutualistic symbiosis with western white pine (*Pinus monticola*) in northern Idaho, USA (Caballero et al., 2023), and *A. luteo‐virens* was considered an ectomycorrhizal symbiont in Qinghai‐Tibet Plateau, China (Xing et al., 2014). The third aspect was that *Armillaria* species suitable for symbiosis with *G. elata* were considered to be low pathogenic and similar in genetic and morphological characteristics, such as *A. cepistipes* and *A. nabsnona*, while *A. mellea* does not fit these characteristics (Guo et al., 2016), which was also supported by some of our unpublished results on rhizomorphs from the mature *G. elata* tubers. We speculated that the relationships between these low pathogenic *Arimillaria* species symbiotic with *G. elata* and adjacent photosynthetic plants could be in the intermediate stage of the evolution toward mycorrhizal symbiosis, on the grounds that ectomycorrhizal symbiosis could originate from saprophytic relationships (Tedersoo et al., 2010) and mycorrhizal cheating could be a common feature of myco‐heterotrophs (Selosse et al., 2010).

## 5 CONCLUSIONS

Based on the results of Illumina® sequencing of the fungal community in early natural *G. elata* tubers and nearby environmental samples, combined with a series of statistical methods, this research demonstrated that the early growth of *G. elata* tubers was related to several different genera of fungi besides *Mycena* and *Armillaria*. These fungi that played central roles were mainly *Sebacina*, *Thelephora*, and *Inocybe*, which were widely confirmed mycorrhizal fungi for different orchids in previous researches. The secondary fungi were mainly *Mycena*, *Auricularia*, and *Cryptococcus*. Although they had little evidence of mycorrhizae in existing researches, they had non‐negligible correlations with those widely confirmed orchid mycorrhizal fungi, and at the same time, their changes in relative abundances from the background environment proved significant and consistent across taxonomic levels. The recruitment process of these secondary symbiotic fungi from the environment should be more selective. In addition, the significant concentration of non‐orchid root‐related fungi in *G. elata* hinted at the possibility of growth‐promoting cheating. Most of these symbiotic fungi were recruited from the environment, especially the rhizosphere soil, but a few were likely to be transmitted vertically from the parents of the host or horizontally from other plants. Indeed, *Armillaria* is not an early symbiont of *G. elata*, and the early symbiotic *Mycena* is not *M. osmundicola* or *M. alphitophora* but is highly possible to be of *M. plumipes* and its sibling species. Our results supported the view that since myco‐heterotrophy was highly possible to be formed in the common ancestor of orchids, common symbiotic fungi among all orchid species should exist. Specific symbiotic fungi, such as *Mycena* which is symbiotic with *G. elata*, could have arisen gradually with the hosts evolution. Although our study provided molecular evidences for symbiotic fungal communities associated with early immature tubers of wild *G. elata*, it is necessary to prove whether some strains are growth‐promoting fungi for the early growth of *G. elata* by using classical isolation and culture techniques in the future.

## AUTHOR CONTRIBUTIONS

**Dong Li:** Data curation (supporting); formal analysis (lead); investigation (supporting); visualization (lead); writing – original draft (lead). **Xiao‐Han Jin:** Investigation (supporting); validation (equal). **Yu Li:** Investigation (supporting); validation (equal). **Yu‐Chuan Wang:** Investigation (supporting); resources (supporting). **Hai‐Yan He:** Investigation (lead); resources (supporting). **Han‐Bo Zhang:** Conceptualization (lead); data curation (lead); funding acquisition (lead); project administration (lead); resources (lead); writing – review and editing (lead).

## CONFLICT OF INTEREST STATEMENT

There is no conflict of interest among the authors of this article.

## CONSENT

All samples were collected by researchers following current Chinese regulations. All local guides employed have been duly rewarded. Neither clinical trials nor patient consents were involved in this research and no material from other sources was used.

## Supporting information


Appendix S1

## Data Availability

The data that support the findings of this study are openly available in the National Centre for Biotechnology Information (NCBI) Sequence Read Archive (SRA) under BioProject ID PRJNA962295, other required information is disclosed in the Supporting Information on the Dryad platform under DOI: https://doi.org/10.5061/dryad.0zpc86749.
