# Supplementary material for: Fungal communities associated with early immature tubers of wild Gastrodia elata
Source: Ecol Evol. 2024 Feb 21;14(2):e11004. doi: 10.1002/ece3.11004 (PMC10881901; doi:10.1002/ece3.11004)
Supplement: Supplementary file 1 — Appendix S1 [file ECE3-14-e11004-s001.pdf]

Table S1 Alpha diversity index calculated for fungal communities in all samples.

samples	observed_otus	shannon	simpson	chao1
HIT1	804	7.20	0.98	812.87
HIT2	965	6.24	0.93	985.24
HIT3	528	6.85	0.98	530.14
HIY1	646	7.09	0.98	646.58
HIY2	661	5.61	0.92	672.44
HIY3	467	3.95	0.79	474.16
LIT1	967	7.35	0.98	971.08
LIT2	766	6.60	0.97	770.68
LIT3	938	5.34	0.80	945.05
LIY1	1401	8.31	0.99	1416.76
LIY2	1028	7.59	0.98	1034.15
LIY3	670	5.99	0.95	677.50
LY1B	98	2.07	0.54	99.11
LY1M	105	1.85	0.44	106.25
LY1P	90	2.36	0.70	90.00
LY2M	75	2.65	0.74	75.33
LY2P	69	1.74	0.44	70.20
LY3M	140	3.00	0.75	141.96
LY3P	58	1.12	0.31	59.88
LYT1	883	7.61	0.99	901.38
LYT3	883	6.51	0.96	897.82
M1IT1	220	4.02	0.88	225.06
M1IT3	268	4.90	0.91	269.07
M1IY1	322	4.14	0.85	326.14
M1IY2	427	4.27	0.89	443.88
M1IY3	230	4.57	0.88	235.20
M2IT2	825	6.66	0.95	830.31
M2IY1	539	5.60	0.94	549.63
M2IY2	739	6.76	0.96	747.30
M2IY3	751	7.23	0.98	755.52
M2Y6B	146	3.61	0.88	150.11
M2Y6M	105	3.95	0.91	107.50
M2Y6P	140	2.95	0.76	142.33
M2Y8B	95	2.71	0.76	95.67
M2Y8M	118	3.61	0.85	119.65
M2Y8P	113	2.92	0.75	113.25
M2YT3	559	5.61	0.94	559.00
MY2B	144	2.80	0.73	146.33
MY2M	156	3.52	0.82	157.47
MY2P	55	0.82	0.18	55.25
MY3B	147	3.74	0.84	147.46
MY3M	121	3.49	0.85	121.08

MY3P	156	3.98	0.90	164.27
MY4M	148	3.74	0.88	149.50
MY4P	110	3.19	0.81	110.30
MYT1	349	2.68	0.48	354.18
MYT2	438	4.88	0.92	443.70
MYT3	418	4.51	0.89	427.02
MYT4	685	5.54	0.94	691.55
MYT5	645	6.17	0.94	647.14
PIT1	616	5.89	0.95	625.36
PIT2	520	5.75	0.94	528.64
PIT3	424	4.81	0.92	429.66
PIY1	642	5.68	0.92	646.60
PIY2	429	3.80	0.74	433.77
PIY3	738	5.85	0.94	756.67
PY1B	128	2.61	0.60	128.71
PY1M	191	3.82	0.86	192.89
PY1P	143	4.19	0.92	144.15
PY2M	171	2.55	0.64	174.88
PY2P	118	2.90	0.79	118.83
PY3M	117	4.06	0.91	118.11
PY3P	103	3.22	0.77	103.25
PYT1	713	6.29	0.97	720.72
PYT2	503	4.65	0.87	520.37
PYT3	576	5.68	0.92	578.35

Table S2 Test of normality, One-way ANOVA and Tukey's multiple comparison of Chao 1 and Shannon diversity indices between sample groups.

Test of normality

	Y	YT	IY	IT
Number of values	28	11	15	12
Chao 1				
D'Agostino & Pearson Test				
K2	3.482	2.428	1.261	0.7844
P value	0.1754	0.2969	0.5323	0.6756
Passed (alpha=0.05)?	Yes	Yes	Yes	Yes
Summary of p-values	ns	ns	ns	ns
Shapiro-Wilk Test				
W	0.9409	0.9576	0.9445	0.9581
P value	0.1164	0.7414	0.4422	0.7562
Passed (alpha=0.05)?	Yes	Yes	Yes	Yes
Summary of p-values	ns	ns	ns	ns
Shannon				
D'Agostino & Pearson Test				
K2	3.482	2.428	1.261	0.7844
P value	0.1754	0.2969	0.5323	0.6756
Passed (alpha=0.05)?	Yes	Yes	Yes	Yes
Summary of p-values	ns	ns	ns	ns
Shapiro-Wilk Test				
W	0.9409	0.9576	0.9445	0.9581
P value	0.1164	0.7414	0.4422	0.7562
Passed (alpha=0.05)?	Yes	Yes	Yes	Yes
Summary of p-values	ns	ns	ns	ns

One-way ANOVA

Chao 1	SS	DF	MS	F	P value
Between groups	4419491	3	1473164	39.50	P<0.0001
Within groups	2312072	62	37291		
Total	6731564	65			
Shannon	SS	DF	MS	F	P value
Between groups	125.3	3	41.75	33.47	P<0.0001
Within groups	77.35	62	1.248		
Total	202.6	65			

Tukey's multiple comparisons test

Chao 1

Test detalis	Mean difference	Significant?	Summary	Adjusted P Value
Y vs. YT	-491.3	Yes	****	<0.0001
Y vs. IY	-532.9	Yes	****	<0.0001
Y vs. IT	-538.7	Yes	****	<0.0001
YT vs. IY	-41.58	No	ns	0.9482
YT vs. IT	-47.42	No	ns	0.9352
IY vs. IT	-5.843	No	ns	0.9998
Shannon				
Test detalis	Mean difference	Significant?	Summary	Adjusted P Value
Y vs. YT	-2.496	Yes	****	<0.0001
Y vs. IY	-2.792	Yes	****	<0.0001
Y vs. IT	-2.997	Yes	****	<0.0001
YT vs. IY	-0.2963	No	ns	0.9086
YT vs. IT	-0.5011	No	ns	0.706
IY vs. IT	-0.2048	No	ns	0.9646

*: Significant differences

ns: No significant differences

Table S3 Adonis analysis illustrating the extent to which different grouping methods explain the differences among samples.

Grouped by	Df	Sum of Sqs	R^2	F	Pr(>F)
Type of source	3	4.3389	0.1474	4.0206	0.001***
Geographical location	3	2.2620	0.0768	2.0960	0.001***
Both	7	4.1229	0.1410	1.6373	0.001***
Residual	52	18.7054	0.6356		
Total	65				
Pairwise comparison based on sample source type					
YT/IY	1	0.9694	0.0888	2.3389	0.001***
YT/IT	1	0.5874	0.0614	1.3737	0.022*
YT/Y	1	1.4526	0.0899	3.6548	0.001***
IY/IT	1	0.9041	0.9041	2.1766	0.001***
IY/Y	1	2.3334	0.0801	2.1766	0.001***
IT/Y	1	1.6562	0.1265	5.9387	0.001***
Pairwise comparison based on geographical location					
PH/MB	1	0.8895	0.8895	2.0386	0.001***
PH/LF	1	0.6638	0.6638	1.4856	0.012*
PH/HZ	1	0.8494	0.8494	1.9901	0.001***
MB/LF	1	0.8901	0.8901	2.0381	0.002**
MB/HZ	1	0.9582	0.9582	2.2719	0.001***
LF/HZ	1	0.7820	0.7820	1.8311	0.002**

*： Significant differences

Distance algorithm: Bray-Curtis dissimilarity

Table S4 Taxonomic relationships, Kruskal-Wallis test results, paired Wilcoxon test results with the other two groups and FUNguild results of the Biomarkers in group Y from the the LEfSe analysis on all fungal phyla.

group			Y								
biomakers	phyla		p_Basidiomycetes			p_Ascomycota					
	classes		c_Agaricomycetes			c_Sordariomycetes				c_Leotiomycetes	
	orders		o_Agaricales				o_Hypocreales	o_Xylariales		o_Helotiales	
	families		f_Mycenaceae					f_Xylariales_fam_Incertae_sedis		f_Helotiaceae	
	genuses			g_Mycena				g_Fusidium		g_Varicosporium	g_Pezoloma
	species				s_Mycena_unclassified				s_Fusidium_unclassified	s_Fontanospora_fusiramosa	
	mean(relative abundance %)		3.4535	3.4535	3.3152	27.3545	14.6170	4.7672	4.7672	2.3991	2.2267
	kruskal.test.p_value		0.0018	0.0018	0.0077	0.0003	0.0016	0.0085	0.0085	0.0021	0.0002
	q_value		0.0047	0.0076	0.0383	0.0005	0.0031	0.0279	0.0416	0.0127	0.0012
vs group	IT (YT)	mean(relative abundance %)	0.8717	0.8714	0.6922	7.1821	4.0809	0.0616	0.0616	0.1038	0.7284

		log2FC	1.99	1.99	2.26	1.93	1.84	6.27	6.27	4.53	1.61
		wilcox.test.p_value	0.0029	0.0029	0.0069	0.0004	0.1323	0.0351	0.0351	1.0000	0.3757
		q_value	0.0068	0.0102	0.0246	0.0009	0.1499	0.0639	0.0802	1.0000	0.4165
		significance	yes	yes	yes	yes	no	yes	yes	no	no
		regulation	up	up	up	up	up	up	up	up	up
	IY	mean(relative abundance%)	0.5673	0.5666	0.5575	8.2866	1.3655	0.1266	0.1266	0.0013	0.0584
		log2FC	2.61	2.61	2.57	1.72	3.42	5.23	5.23	10.85	5.25
		wilcox.test.p_value	0.0049	0.0049	0.0057	0.0006	0.0002	0.0042	0.0042	0.0005	0.0007
		q_value	0.0122	0.0158	0.0273	0.0012	0.0006	0.0149	0.0223	0.0037	0.0034
		significance	yes	yes	yes	yes	yes	yes	yes	yes	yes
		regulation	up	up	up	up	up	up	up	up	up
FUNGuild			Probable leaf saprotroph-Plant pathogen-undifined					Probable undefined saprotroph			

	saprotroph-woodsaprotroph					

Table S5 Taxonomic relationships, Kruskal-Wallis test results, paired Wilcoxon test results with the other two groups and FUNguild results of the Biomarkers in group IT(YT) from the LEfSe analysis on all fungal phyla.

group		IT(YT)				
biomakers	phyla	p_Basidiomycota				
	classes	c_Agaricomycetes				
	orders	o_Russulales		o_Boletales		o_Agaricales
	families	f_Russulaceae		f_Rhizopogonaceae		f_Hygrophoraceae
	genuses	g_Lactarius			g_Rhizopogon	
	species		s_Arcangeliella_borziana			
	mean(relative abundance %)	3.3380	3.3362	2.6185	2.6185	1.6666
	kruskal.test.p_value	0.0044	0.0007	0.0486	0.0486	0.0012
	q_value	0.0159	0.0052	0.0862	0.1092	0.0034
Y	mean(relative abundance %)	0.0108	0.0002	0.0013	0.0013	0
	log2FC	-8.27	-14.03	-10.98	-10.98	-Inf
	wilcox.test.p_value	0.0007	0.0001	0.0264	0.0264	0.0003
	q_value	0.0034	0.0012	0.0439	0.0534	0.001
	significance	yes	yes	yes	yes	yes
	regulation	up	up	up	up	up
IY	mean(relative abundance %)	0.5271	0.5271	0.2353	0.2353	0.0013
	log2FC	2.66	2.66	3.48	3.48	10.32

	wilcox.test.p_value	0.077	0.077	0.4642	0.4642	0.147
	q_value	0.3686	0.4745	0.6101	0.5956	0.3954
	significance	no	no	no	no	no
	regulation	up	up	up	up	up
FUNGuild		Highly probable ectomycorrhizal				Probable Ectomycorrhizal-undefined saprotroph

Table S6 Taxonomic relationships, Kruskal-Wallis test results, paired Wilcoxon test results with the other two groups and FUNguild results of the Biomarkers in group IY from the LEfSe analysis on all fungal phyla.

<table>
<tr><td colspan="2">group</td><td colspan="7">IY</td></tr>
<tr><td rowspan="10">biomarkers</td><td>phyla</td><td colspan="7">p_Ascomycota</td></tr>
<tr><td>classes</td><td colspan="4">c_Leotiomycetes</td><td>c_Dothideomycetes</td><td colspan="2">c_Pezizomycotina_Incertae_sedis</td></tr>
<tr><td>orders</td><td colspan="4">o_Helotiales</td><td></td><td colspan="2">o_Pezizomycotina_Incertae_sedis</td></tr>
<tr><td>families</td><td colspan="2">f_Sclerotiniaceae</td><td colspan="2">f_Dermateaceae</td><td></td><td colspan="2">f_Pezizomycotina_Incertae_sedis</td></tr>
<tr><td>genuses</td><td>g_Sclerotiniaceae_unclassified</td><td>g_Sclerotiniaceae_unclassified</td><td colspan="2">g_Pezicula</td><td></td><td colspan="2">g_Coleophoma</td></tr>
<tr><td>species</td><td></td><td>s_Sclerotiniaceae_unclassified</td><td></td><td>s_Pezicula_alnicola</td><td></td><td></td><td>s_Coleophoma_empetri</td></tr>
<tr><td>mean(relative abundance %)</td><td>4.8022</td><td>4.8022</td><td>3.1922</td><td>3.1901</td><td>11.4257</td><td>3.4707</td><td>3.4707</td></tr>
<tr><td>kruskal.test.p_value</td><td>0.0001</td><td>0.0001</td><td>0.0004</td><td>0.0002</td><td>0.0001</td><td>0.0001</td><td>0.0001</td></tr>
<tr><td>q_value</td><td>0.0006</td><td>0.0010</td><td>0.0021</td><td>0.0019</td><td>0.0002</td><td>0.0006</td><td>0.0010</td></tr>
<tr style="display:none"><td colspan="8"></td></tr>
<tr><td rowspan="3">Y</td><td>mean(relative abundance %)</td><td>0.0129</td><td>0.0129</td><td>0.0051</td><td>0.0002</td><td>5.5682</td><td>0</td><td>0</td></tr>
<tr><td>log2FC</td><td>-8.54</td><td>-8.54</td><td>-9.29</td><td>-13.96</td><td>-1.04</td><td>-Inf</td><td>-Inf</td></tr>
<tr><td>wilcox.test.p_value</td><td>0.0000</td><td>0.0000</td><td>0.0001</td><td>0.0000</td><td>0.0002</td><td>0</td><td>0</td></tr>
</table>

	q_value	0.0000	0.0000	0.0006	0.0000	0.0005	0	0
	significance	yes	yes	yes	yes	yes	yes	yes
	regulation	up	up	up	up	up	up	up
IT	mean(relative abundance %)	1.0418	1.0418	0.0551	0.0536	2.5512	0.0123	0.0123
	log2FC	-2.2	-2.2	-5.86	-5.9	-2.16	-8.14	-8.14
	wilcox.test.p_value	0.2915	0.2915	0.2452	0.2339	0.0073	0.1147	0.1147
	q_value	0.5162	0.4969	0.5162	0.4969	0.0353	0.4144	0.4969
	significance	no	no	no	no	yes	no	no
	regulation	up	up	up	up	up	up	up
FUNGuild		Probable plant pathogen					Probable undefined saprotroph	

Table S7 Taxonomic relationships, Kruskal-Wallis test results, paired Wilcoxon test results with the other two groups and FUNguild results of the Biomarkers in group Y from the LEfSe. Analysis on Basidiomycetes.

group			Y			
biomakers	classes		c_Tremellomycetes			c_Agaricomycetes
	orders		o_Tremellales			o_Auriculariales
	families		f_Tremellaceae			f_Auriculariales_unclassified
	genuses			g_Cryptococcus		g_Auriculariales_unclassified
	species				s_Cryptpcoccus_podzolicus	s_Auricularia_unclassified
	mean		32.0777	32.0777	19.615	7.1174
	kruskal.test.p_value		0.002	0.0016	0.0001	0.0286
	q_value		0.0075	0.0102	0.0019	0.1078
vs group	YT	mean	5.5499	5.5478	2.69	0.2473
		log2FC	2.53	2.53	2.86	4.85
		wilcox.test.p_value	0.005	0.005	0.006	0.0154
		q_value	0.0141	0.0188	0.0314	0.0589
		significance	yes	yes	yes	yes
		regulation	up	up	up	up
	IT	mean	9.634	9.5995	6.4199	0.0438
		log2FC	1.74	1.74	1.61	7.34
		wilcox.test.p_value	0.0061	0.0061	0.0086	0.9036
		q_value	0.0187	0.0222	0.0386	0.9064
		significance	yes	yes	yes	no
		regulation	up	up	up	up
	IY	mean	9.4824	8.2277	1.299	0.0776
		log2FC	1.76	1.96	3.92	6.52
		wilcox.test.p_value	0.0087	0.0037	0	0.6672
		q_value	0.0263	0.0193	0	0.6921
		significance	yes	yes	yes	no
		regulation	up	up	up	up

FUNGuild	Possiable Animal Pathogen-Endophyte-Epiphyte-Undefined Saprotroph	

Table S8 Taxonomic relationships, Kruskal-Wallis test results, paired Wilcoxon test results with the other two groups and FUNguild results of the Biomarkers in group YT from the LEfSe. Analysis on Basidiomycetes.

group			YT						
biomakers	classes		c_Agaricomycetes						
	orders		o_Russulales			o_Cantharellales			
	families		f_Russulaceae				f_Clavulinaceae		
	genuses		g_Russula					g_Clavulina	
	species			s_Russula_senecis	s_Russula_nigricans				s_Clavulina_unclassified
	mean		37.1568	6.6093	17.2127	6.1073	5.6272	5.6243	5.6243
	kruskal.test.p_value		0.0055	0	0.0006	0	0.0008	0.001	0.0004
	q_value		0.0266	0	0.0073	0	0.0039	0.0073	0.0052
vs group	Y	mean	3.2533	0.0403	1.8069	0.0544	0.0544	0.0544	0.0544
		log2FC	-3.51	-7.36	-3.25	-6.81	-6.69	-6.69	-6.69
		wilcox.test.p_value	0.0044	0.0003	0.0016	0	0.0001	0.0001	0.0001
		q_value	0.0177	0.0051	0.0163	0	0.0008	0.0016	0.0036
		significance	yes	yes	yes	yes	yes	yes	yes
		regulation	up	up	up	up	up	up	up
	IT	mean	10.4538	0	0.7374	0.9095	0.4745	0.4716	0.4716
		log2FC	-1.83	-Inf	-4.54	-2.75	-3.57	-3.58	-3.58

		wilcox.test.p_value	0.0635	0.00	0.0022	0.4237	0.0888	0.068	0.068
		q_value	0.531	0.48	0.4833	0.7538	0.6589	0.531	0.4833
		significance	no	yes	yes	no	no	no	no
		regulation	up	up	up	up	up	up	up
	IY	mean	2.6452	0	0.6685	2.2817	0.1151	0.1151	0.0316
		log2FC	-3.81	-Inf	-4.69	-1.42	-5.61	-5.61	-7.48
		wilcox.test.p_value	0.0027	0.0016	0.0009	0.7753	0.0086	0.0086	0.0021
		q_value	0.0525	0.0842	0.0743	0.8764	0.1015	0.1315	0.0937
		significance	yes	yes	yes	no	yes	yes	yes
		regulation	up	up	up	up	up	up	up
FUNGuild			Highly Probable Ectomycorrhizal					Highly Probable Ectomycorrhizal	

Table S9 Taxonomic relationships, Kruskal-Wallis test results, paired Wilcoxon test results with the other two groups and FUNguild results of the Biomarkers in group IT from the LEfSe. Analysis on Basidiomycetes.

group			IT							
biomarkers	classes		c_Agaricomycetes							
	orders		o_Agaricales					o_Boletales	o_Russulales	
	families		f_Hygrophoraceae	f_Clavariaceae			f_Agaricales_unclassified		f_Russulaceae	
	genuses				g_Clavulinopsis				g_Lactarius	
	species					s_Clavulinopsis_helvola				s_Arcangeliella_borziana
	mean		4.3757	9.8548	7.9533	7.0216	7.1274	6.3323	5.9079	5.9051
	kruskal.test.p_value		0.0015	0.0001	0	0	0	0	0.0111	0.0013
	q_value		0.0061	0.0006	0	0	0	0	0.0455	0.0134
vs group	Y	mean	0	1.5835	0.2769	0	0.3928	0.0678	0.3129	0.0048
		log2FC	-Inf	-2.64	-4.84	-Inf	-4.18	-6.55	-4.24	-10.26
		wilcox.test.p_value	0.0003	0.0004	0	0	0	0	0.0012	0.0001
		q_value	0.0016	0.0021	0	0	0	0	0.0081	0.0029
		significance	yes	yes	yes	yes	yes	yes	yes	yes
		regulation	up	up	up	up	up	up	up	up
	YT	mean	3.2252	6.2846	4.2374	0.2583	1.34	1.0029	0.6162	0.0365
		log2FC	0.44	0.65	0.91	4.76	2.41	2.66	3.26	7.34

		wilcox.test.p_value	0.6835	0.902	0.498	0.2534	0.3248	0.0267	0.2969	0.0772
		q_value	0.8822	0.9535	0.6751	0.4833	0.689	0.7201	0.531	0.4833
		significance	no	no	no	no	no	yes	no	no
		regulation	up	up	up	up	up	up	up	up
	IY	mean	0.0217	0.5812	1.0051	0.5812	1.3903	5.0686	1.0324	1.0324
		log2FC	7.66	3.59	2.98	3.59	2.36	0.32	2.52	2.52
		wilcox.test.p_value	0.1837	0.1205	0.0242	0.1205	0.1712	0.3797	0.1635	0.1635
		q_value	0.501	0.4898	0.2466	0.4898	0.4891	0.5056	0.5333	0.4898
		significance	no	no	yes	no	no	no	no	no
		regulation	up	up	up	up	up	up	up	up
FUNGuild			Probable Ectomycorrhizal-Undefined Saprotroph	Probable Lichenized-Undefined Saprotroph	Probable Undefined Saprotroph		Possible Bryophyte Parasite-Dung Saprotroph-Ectomycorrhizal-Fungal Parasite-Leaf Saprotroph-Plant Parasite-Undefined Saprotroph-Wood		Highly Probable Ectomycorrhiza	

					Saprotroph			

Table S10 Taxonomic relationships, Kruskal-Wallis test results, paired Wilcoxon test results with the other two groups and FUNguild results of the Biomarkers in group IY from the LEfSe. Analysis on Basidiomycetes.

group			IY								
biomarkers	classes		c_Agaricomycetes				c_Microbotryomycetes			c_Tremellomycetes	c_Basidiomycota_unclassified
	orders		o_Atheliales					o_Sporidiobolales		o_Tremellales	
	families			f_Atheliaceae					f_Sporidiobolales_Incertae_sedis	f_Tremellales_Incertae_sedis	
	genuses				g_Fibulorhizoctonia						
	species					s_Fibulorhizoctonia_sq_TMB					
	mean		5.8237	5.8237	5.7297	5.7297	5.7363	5.5635	5.5607	4.1449	5.5492
	kruskal.test.p_value		0.0003	0.0003	0.0003	0.0003	0	0	0	0	0
	q_value		0.0011	0.0017	0.0029	0.005	0	0	0	0	0
vs group	Y	mean	0.114	0.114	0	0	0.9129	0.4775	0.4775	0	0.758
		log2FC	-5.67	-5.67	-Inf	-Inf	-2.65	-3.54	-3.54	-Inf	-2.87

		wilcox.test.p_value	0.00002	0.00002	0.00004	0.0004	0	0	0	0	0
		q_value	0.00007	0.00014	0.00032	0.0048	0	0	0	0	0
		significance	yes	yes	yes	yes	yes	yes	yes	yes	yes
		regulation	up	up	up	up	up	up	up	up	up
	YT	mean	0.24	0.24	0.0013	0.0013	0.2641	0.1579	0.1579	0.0737	0.75
		log2FC	4.6	4.6	12.11	12.11	4.44	5.14	5.14	5.81	2.88
		wilcox.test.p_value	0.8127	0.8127	0.0583	0.0583	0.0006	0.0004	0.0004	0.0001	0.0017
		q_value	0.8764	0.8676	0.3794	0.4422	0.0035	0.0057	0.0157	0.0118	0.0064
		significance	no	no	no	no	yes	yes	yes	yes	yes
		regulation	up	up	up	up	up	up	up	up	up
	IT	mean	1.7533	1.7533	0	0	0.1777	0.1612	0.1612	0.0243	1.8114

		log2FC	-1.73	-1.73	-Inf	-Inf	-5.01	-5.11	-5.11	-7.41	-1.62
		wilcox.test.p_value	0.3852	0.3852	0.0158	0.0158	0.0004	0.0003	0.0003	0	0.0112
		q_value	0.5056	0.5851	0.1989	0.3117	0.0049	0.0042	0.009	0	0.0261
		significance	no	no	yes	yes	yes	yes	yes	yes	yes
		regulation	up	up	up	up	up	up	up	up	up
FUNGuild				Possiable Ectomycorrhizal-Lichen Parasite-Lichenized-Plant Pathogen							

Table S11 110 Possible growth-promoting fungal ASVs in group Y.

ASV_ID	Rename_as
2b8ef4995b569aebd7e43752d2e28a3b	s_Mycena_unclassified_01
77408907395b8d3f4c554cff61b0c9eb	s_Mycena_plumipes_01
88890c90108440d98792b31e86c13bb8	s_Mycena_unclassified_02
907ac3e2160acaecd0dcbdd468592f00	s_Mycena_unclassified_03
0a082c14f402b6293163c91a67c6819e	s_Mycena_plumipes_02
3aff4483c0de0ecffe6d064d29a461b1	s_Mycena_unclassified_04
7b4622352e6e1239c037f5c407672e7b	s_Mycena_unclassified_05
64ba69305664529ddffb8239b928ad27	s_Russula_nigricans_01
61ed9f880eb08fa516e8e7370639c6af	s_Russula_inamoena
1b0b74eed20ed2c23718d4f879143848	s_Russula_senecis
5c9d71bedb0d135aa8aefcc38039c3b2	s_Russula_nigricans_02
a7bbd3f1e65023ba09464693ef74f5db	s_Russula_nigricans_03
6281b0a1cfdd6baaa1c5fd1b2938bcac	s_Russula_cyanoxantha_01
4e3ecab6883c7c14f95283e9b9b482f9	s_Russula_cyanoxantha_02
29852d0e857edba5eae1caf8d105d2e1	s_Russula_unclassified_01
f558239f2b592bcce8303588d02a7f42	s_Russula_cyanoxantha_03
043b52760d392fa417343947cad8e3f3	s_Russula_unclassified_02
cbd0fb2848cc4fb3e92708379bdf8ce7	s_Russula_earlei
6907b4581a7ab2e969f64c4a2c52ee6c	s_Russula_laccata_01
cadfca72c1a6f84db121dc0218c1abda	s_Russula_laccata_02
26f06b72ec3ae03246af9c1b69f12c6f	s_Russula_cascadensis
96aaf60ad84665a652724f27834f91d0	s_Russula_nauseosa
90edc14993f90fc38e5654389d57b6d5	s_Inocybe_unclassified_01
bda1a7a517d982b7e79d6144c3540041	s_Inocybe_unclassified_02
30b98a8cfeb2b7811990e21ec687b1c8	s_Inocybe_mimica
a8280354aa14c15db86f5fbbe999e770	s_Inocybe_hirtella
891e67193730cb19162b14e9e0fb230b	s_Psathyrella_candolleana_01
3b0492e4b149ab45d09f541de4799266	s_Psathyrella_candolleana_02
13f0d14a0d3eb851c38be968d08e48e3	s_Clavulina_unclassified_01
7514f83370509dc568e6c8e9e42e3bdf	s_Clavulina_unclassified_02
518596130a9a584fb65024dca2f8c1f8	s_Sebacina_incrustans_01
3a3c9e5f0288e602906fa1eeaffaceb2	s_Sebacina_unclassified_01
e04dd46ce06a03c5e119ec33b799b9e1	s_Sebacina_incrustans_02
1481f8ee70c1f8998273fbecb8f8cfe7	s_Sebacina_incrustans_03
cd30d22943175e6570a3ef34ce703e94	s_Sebacina_unclassified_02
bdfaccca3ade1659f948c1aef93ee3a4	s_Sebacina_incrustans_04
0cd026ba8ae41aefdb23b0e6c853d109	s_Sebacina_incrustans_05
b0debc753a2dee41589e04436a0f8e6c	s_Sebacina_incrustans_06
e241c3fc469251db4b500a964947e935	s_Sebacina_incrustans_07
fbb796c4a20ac9caa04899c5163d33e7	s_Sebacina_incrustans_08
c1cdd2770bb0da27c473ef04a5013bb8	s_Sebacinales_unclassified_03
f2b7f0ccf09617285136059b6502d132	s_Sebacina_vermifera_01

1d7d1625594a29ddeae3fc4787fa4adf	s_Sebacina_vermifera_02
d7b0b4919456b50130f5abb228706dbf	s_Sebacina_incrustans_09
9fb81d937b56b5eee94d2ec5385f9c00	s_Sebacina_incrustans_10
8dcba08bae855bd1f0271781201c2a29	s_Sebacinales_unclassified_04
2b5a721b1af0fb598643b47aecd6f6e6	s_Thelephora_unclassified_01
11f01b9db1d23a6266f572c717e744c0	s_Thelephora_unclassified_02
eaed2c943bf6228245d831e4606ce3c4	s_Thelephora_unclassified_03
a0dfaba36274d6f4cee0957a6bc46056	s_Thelephora_unclassified_04
84a4f33832e9609dd839fb9fb32e9717	s_Pseudotomentella_tristis_01
8f5f63f657094c7d9623b96b1ce06b3b	s_Pseudotomentella_tristis_02
3b3a31dfac617a4bc9652849364338f5	s_Pseudotomentella_tristis_03
24b1f54051f4f2888c9cbb4373f6e2d3	s_Pseudotomentella_atrofusca
c946adf8c2356d7ab4b84b88249793a1	s_Pseudotomentella_tristis_04
2eeaa70e47d92b0ed88b748aa68fc1bb	s_Pseudotomentella_tristis_05
0099c62a17cd2e92b3defd093e92ffd9	s_Tomentella_sublilacina_01
1bf5eb1527bb349cf2dcfc90e0bb35ca	s_Tomentella_sublilacina_02
f9b1994e1ffeb449f5c8645a87b2fda6	s_Tomentella_ellisii_01
85555f6895b73556cb4c00b9f1e3480d	s_Tomentella_unclassified_01
0e844da4d84dfc8a7e9c3c6d3954d000	s_Tomentella_unclassified_02
4233668c51da57d3e51e4499b36e6507	s_Tomentella_unclassified_03
1234c751e9b8c894f95cd843dd2defb4	s_Tomentella_unclassified_04
94265c68969a032a468ca9b4ab596723	s_Tomentella_unclassified_05
2bbf7d786bbf7e9304d0c34016c6da5d	s_Tomentella_stuposa
48ae00e73329cfec103c5a3843d5b172	s_Tomentella_unclassified_06
99e17ec0195496a28e337c15befa8307	s_Tomentella_ellisii_02
0e2284f141fc440a2588437f1ccf88ee	s_Tomentella_badia
ffc39fcffa97097f4ef41c0ef11bb3e0	s_Tomentella_unclassified_07
ece73c77d7bb5dd1006f616f09a8a97c	s_Tomentella_unclassified_08
102e5d265556f78b903a848109238bee	s_Tomentella_unclassified_09
deb9b6a3dbe3b30235f2397b37300489	s_Tuber_unclassified_01
0d615fb5ec279b7d70f532befb17347e	s_Tuber_unclassified_02
fd497d7eb59efd3edb39047b23dbf3e2	s_Tuber_unclassified_03
e117691d657fd1101a969d9c44e16740	s_Cryptococcus_podzolicus_01
b61628f67cb9013d04d78fe17604d155	s_Cryptococcus_terricola_01
3806cb3ee8a28e94922c09ac5ceb3643	s_Cryptococcus_terreus
9989a13264344fa2e675df265229a352	s_Cryptococcus_podzolicus_02
3b3ab58305c91b0938210c35b04fbff2	s_Cryptococcus_podzolicus_03
3decf0c64797b39ac441100db58936a8	s_Cryptococcus_podzolicus_04
9fafbd34b5b6ad677b31122df1b1a82c	s_Cryptococcus_terricola_02
a0a28bbde5ea72dde3bb6c70b6b50dce	s_Cryptococcus_unclassified_01
ca74b3c270fd38ff4476cb5c00e215ee	s_Cryptococcus_podzolicus_05
0e6126664b5bcc7d5f776a76743791f8	s_Cryptococcus_podzolicus_06
41864a9fa7a3d92c3b3499d18cf8223d	s_Cryptococcus_unclassified_02
9ea0fc85aed45e981b6ed547282c489a	s_Cryptococcus_podzolicus_07

9696e9155c4d69e1999cf018aab5f8e9	s_Cryptococcus_podzolicus_08
82c7b03e5fe4b88cc6ab041863bc2eb0	s_Cryptococcus_podzolicus_09
0b4ab1aecc9d2fdd6d595ec4ba45983d	s_Cryptococcus_magnus
8530df0db30e5ab677edcb29e4b4fea3	s_Cryptococcus_podzolicus_10
104fbd8430a8f964678618a8dfd85581	s_Cryptococcus_podzolicus_11
812562a8164f70100fddeb02edc83274	s_Cryptococcus_podzolicus_12
6d68debf089a135857ebef4d1aef8d3f	s_Cryptococcus_podzolicus_13
0400fa62f81c837d2901d8a30237bfef	s_Cryptococcus_unclassified_03
1e974af7267c7d7135f9e819ef9dac29	s_Cryptococcus_unclassified_04
82daaeede429048d53f7684e85b38273	s_Cryptococcus_unclassified_05
2fb00ba623af71b72e4d701ea679889f	s_Cryptococcus_unclassified_06
53dcd2b7d83830b388813f6e4e790863	s_Auricularia_unclassified_01
de34cdb0e2ac3b5fcc16453b2f56cd9a	s_Auricularia_unclassified_02
745a378256d8122cce31c87d4a043b10	s_Auricularia_unclassified_03
8edd0a01b4a0d3364e7004648d9352ff	s_Auricularia_auricula_judae
3a969615ef7ab51ac4a34150877fdf87	s_Scopuloides_hydnoides_01
903a8fced1c7c82ede22091c2372b823	s_Scopuloides_hydnoides_02
43d5fb3e82d13b0280decc80dcf9df2b	s_Ramariopsis_unclassified_01
1fdfb8ef5b9085d6aba233e30aa28d8d	s_Ramariopsis_unclassified_02
5aa99b05f8e9f95a02df525cd551e0fc	s_Ramariopsis_unclassified_03
baa01def757a08bcdf85e15defe94db6	s_Ramariopsis_unclassified_04
5820adb612d5d045364b0f4c9d7dc3d5	s_Flagelloscypha_minutissima_01
58c9942cbd5f55e26743262fd36c58f2	s_Flagelloscypha_minutissima_02
2854018b969d33a8b47154d75dd5c2f5	s_Flagelloscypha_minutissima_03

Table S12 Sequences of the Top 11 possible growth-promoting fungal ASVs (presented in fasta format).

>s_Mycena_unclassified_01
AACGCACCTTGCGCCCTTTGGTATTCCGAAGGGCATGCCTGTTTGAGTGTC
ATTAAATTATCAACCTTGTTCGCTTTTACGAGCTTGAGCGAGGCTTGGATG
TGAGGGCTTGCTGGCTTCCTTCAGTGGATGGTCTGCTCCCTTTAAATGCAT
TAGTGGGATCTCTTGTGGACCGTCACTTGGTGTGATAATTATCTATGCCAT
TTGACTGTGAAGCAAAATTATGGGAACCTGCT
>s_Pseudotomentella_tristis_01
AACGCACCTTGCGCTCCTTGGTATTCCGAGGAGCATGCCTGTTTGAGTGTC
ATGAAATTCTCAACTGCCTTTTAGCATTTATGTTAAGGGTGAGGTTGGAGT
TGGAGGATAATTGCTGGCGCAACTGAGGTTGCTTGTTGGCTCCTCTTGAAT
GGATGAGCTTTTCTGATGGGATCCATAGCTCAGTAACACTAATGTGATAAT
TATCTACGTTAGATGTGAAGAGGCTCCTACAG
>s_Pseudotomentella_tristis_02
AACGCACCTTGCGCTCCTTGGTATTCCGAGGAGCATGCCTGTTTGAGTGTC
ATGAAATTCTCAACTGCCTTTTAGCATTTATGTTAAGGGTGAGGTTGGAGT
TGGAGGATAATTGCTGGCGCAACTGAGGTTGCTTGTTGGCTCCTCTTGAAT
GGATGAGCTTTTCTGATGGGATCCATAGCTCAGTAACACTAATGTGATAAT
TATCTACGTTAGATGTGACGAGGCTCCTACAG
>s_Auricularia_unclassified_01
AACGCACCTTGCGCTCCTTGGTATTCCGAGGAGCATGCCTGTTTGAGTGTC
ACGTGAAACCCTCACCCCTGCGATGTCAAAGTCGTGGACGGTGGATTTGG
ACGCTGCCGTTGTTGGCTCGTCTCGAAATGCATTAGCCGTGCGCGTCGCGC
CAGCGCCTCTCGGTGTGATAATTATCTACGCCGTTCAGAGTGCCAGTGCCC
AAGACCGTGGCTTATAGCCGTCTTCGGACAATT
>s_Cryptococcus_podzolicus_01
AACGCACCTTGCGCCCTCTGGTATTCCGGAGGGCATGCCTGTTTGAGTGTC
ATGTAGACTCAATCCCTCGGGTTTCCGAGGAGATTGGACTTGGGTGTTGCC
GCTCTGCCGGCTCGCCTTAAAAGACTTAGCGGGATAGCACCGTAGTCGGC
GTAATAAGTTTCGTCGGTGAAGGTTGTGATGACTGCTTACAATCGCCCTCG
GGCAATTTTTGACTCTGACCTCAAATCAGGTAG
>s_Cryptococcus_terricola_01
AACGCACCTTGCGCTCTTTGGTATTCCGAAGAGCATGCCTGTTTGAGTGTC
ATGAAAATATCAACCTTGACTTGGGTTTAGTGCTCTTGTCTTGGCTTGGAT
TTGGCTGTTTGCCGCTCGAAAGAGTCGGCTCAGCTTAAAAGTATTAGCTG
GATCTGTCTTTGAGACTTGGTTTGACTTGGCGTAATAAGTTATTTCGCTGA
GGACAATCTTCGGATTGGCCGAGTTTCTGGGAC
>s_Mycena_plumipes_01
AACGCACCTTGCGCCCTTTGGTATTCCGAAGGGCATGCCTGTTTGAGTGTC
ATTAAATTATCAACCTTGTTCGCTTTTACGAGCTTGAGCGAGGCTTGGATG
TGAGGGCTTGCTGGCTTCCTTCAGTGGATGGTCTGCTCCCTTTAAAGGCAT

TAGTGGGATCTCTTGTGGACCGTCACTTGGTGTGATAATTATCTATGCCAT
TTGACTGTGAAGCAAAATTATGGGAACCTGCT
>s_Sebacina_unclassified_01
AACGCACCTTGCACCCTTTGGTATTCCAAAGGGTATGCTCGTTTGAGTGTC
ATTGTACTCTCACACTCTCTGATCTTAGGATTGGGAGTGGTGGATTTGGGT
GTTGCGGCTTCACTGTGGCTCACCTAAAATGCATTAGTGCAACTCTTGACT
AAACCATAGTATGGCATGATAAGTATCTTCGCCAGCACCTCTCCGCAGGG
TGGTCAATCGAGAGCTCTGTGCCTCTAACTGTC
>s_Sebacina_unclassified_02
AACGCACCTTGCACCCTTTGGTATTCCAAAGGGTATGCTCGTTTGAGTGTC
ATTGTACTCTCACACTCTCTGATCTTAGGATTGGGAGTGGTGGATTTGGGT
GTTGCGGCTTCACTGTGGCTCACCTAAAATGCATTAGTGCAACTCTTGACT
AAACCATAGTATGGCATGATAAGTATCTTTGCCAGCACCTCTCCGCAGGG
TGGTCAATCAAGAGCTCTGTGCTGCTAACTGTC
>s_Scopuloides_hydnoides_01
AACGCACCTTGCGCTCCTTGGTATTCCGAGGAGCATGCCTGTTTGAGTGTC
ATGGTATTCTCAACTCTACAAAGCTTTTGCAATGAAGAGTTGGATTTGGAG
GCTTGTGTTGGCTCGAAAGAGTTGACTCCTCTGAAATGCATTAGTGTGAAC
CTTTACGGATCGCCTCCAGTGTGATAATGTCTACGCTGTAGTGTTGAAGTA
TTTCTAGTGTTCATGCTTCTAATCGTCCTCTT
> s_Scopuloides_hydnoides_02
AACGCACCTTGCGCTCCTTGGTATTCCGAGGAGCATGCCTGTTTGAGTGTC
ATGGTATTCTCAACTCTACAAAGCTTTTGCAATGAAGAGTTGGATTTGGAG
GCTTGTGTTGGCTCGAAAGAGTTGACTCCTCTGAAATGCATTAGTGTGAAC
CTTTACGGATCGCCTCCAGTGTGATAATGTCTACGCTGTGGTGTTGAAGTA
TTTCTAGTGTTCATGCTTCTAACCGTCCTCTT

Figure S1 Specific geographical location of the four study site: Panhe, Haizi, Mangbu and Lianfeng.

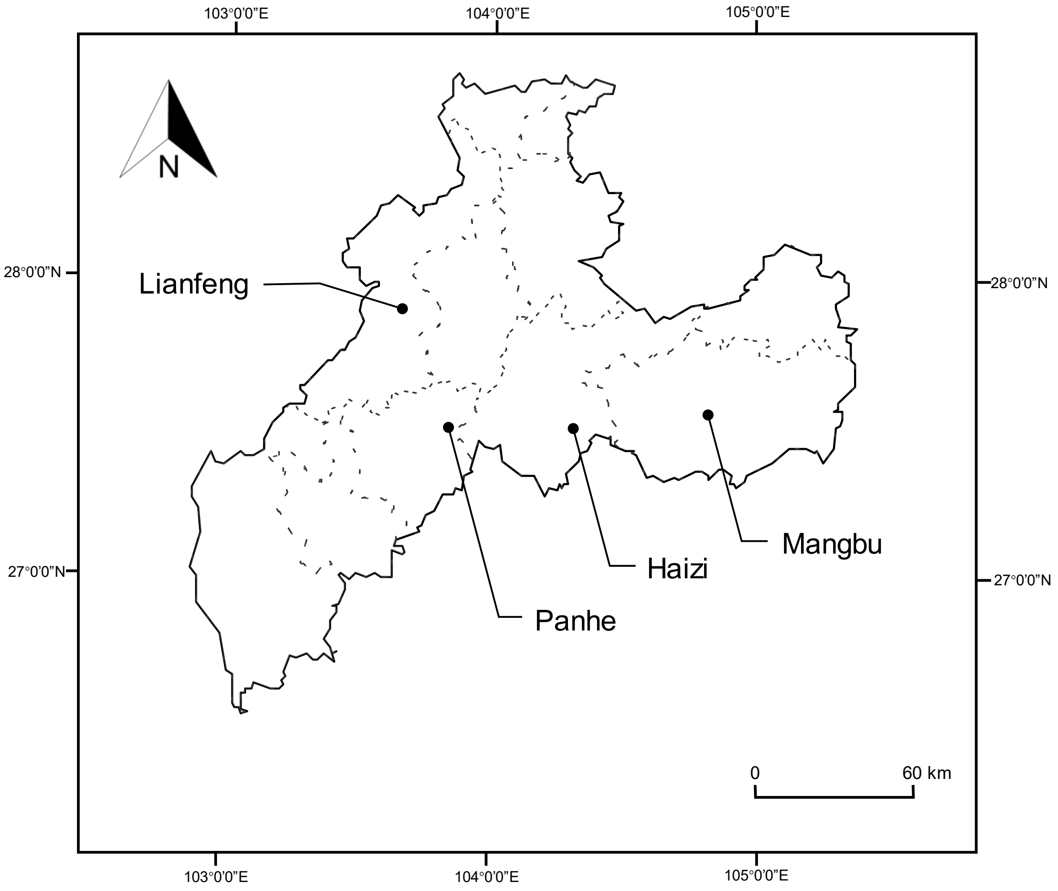

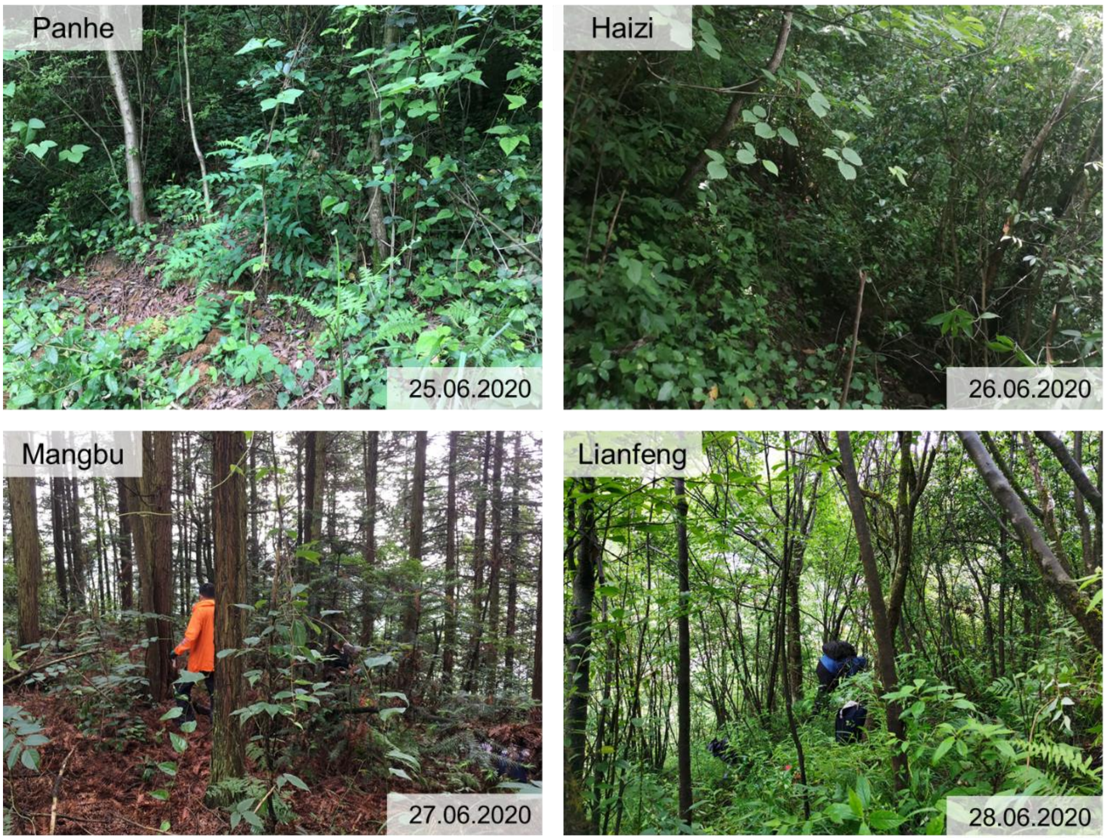

Figure S2 Field photographs of the study site.

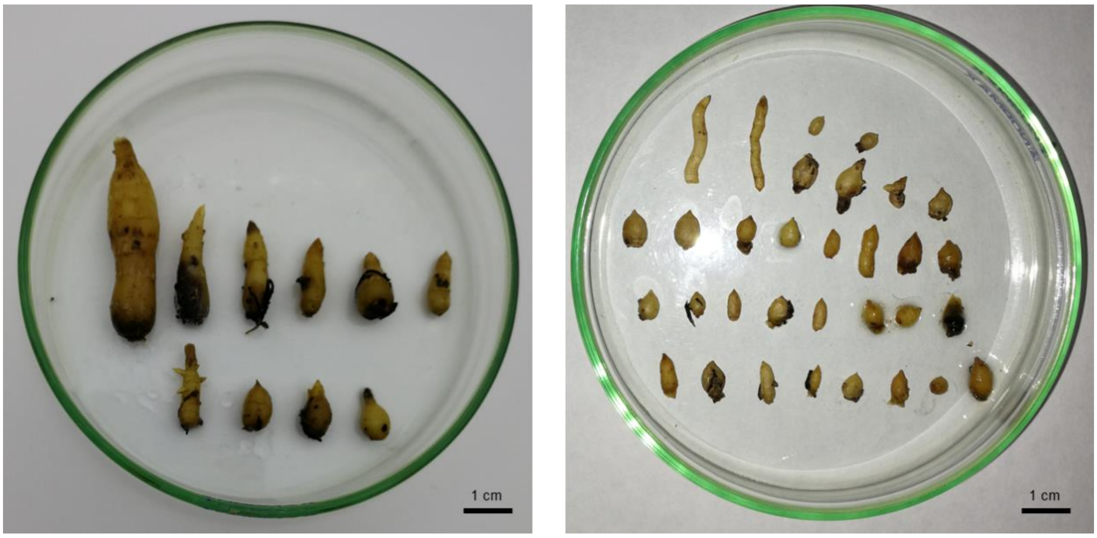

Figure S3 Wild *Gastrodia elata* tubers isolated from soil and litter, these black patches were assumed to be diseased spots.

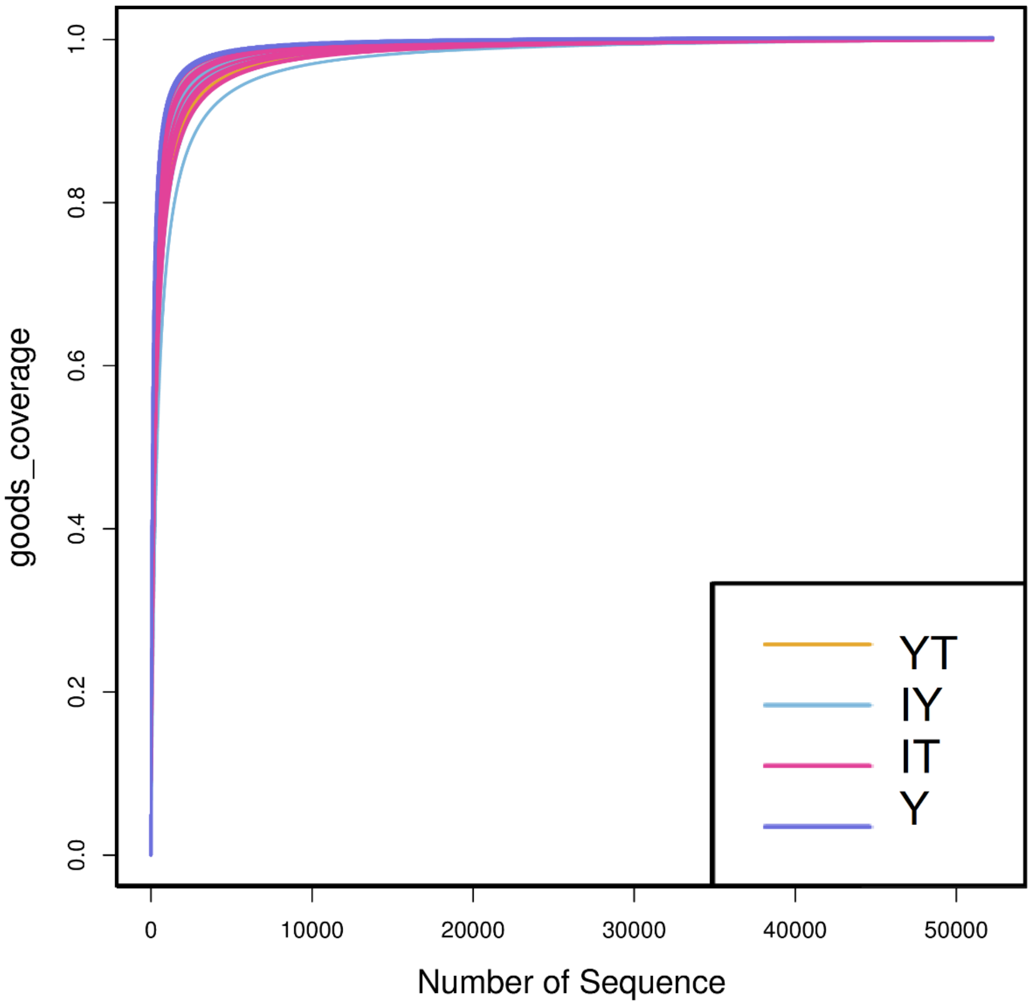

Figure S4 Rarefaction curves of Good’s coverage indexes.

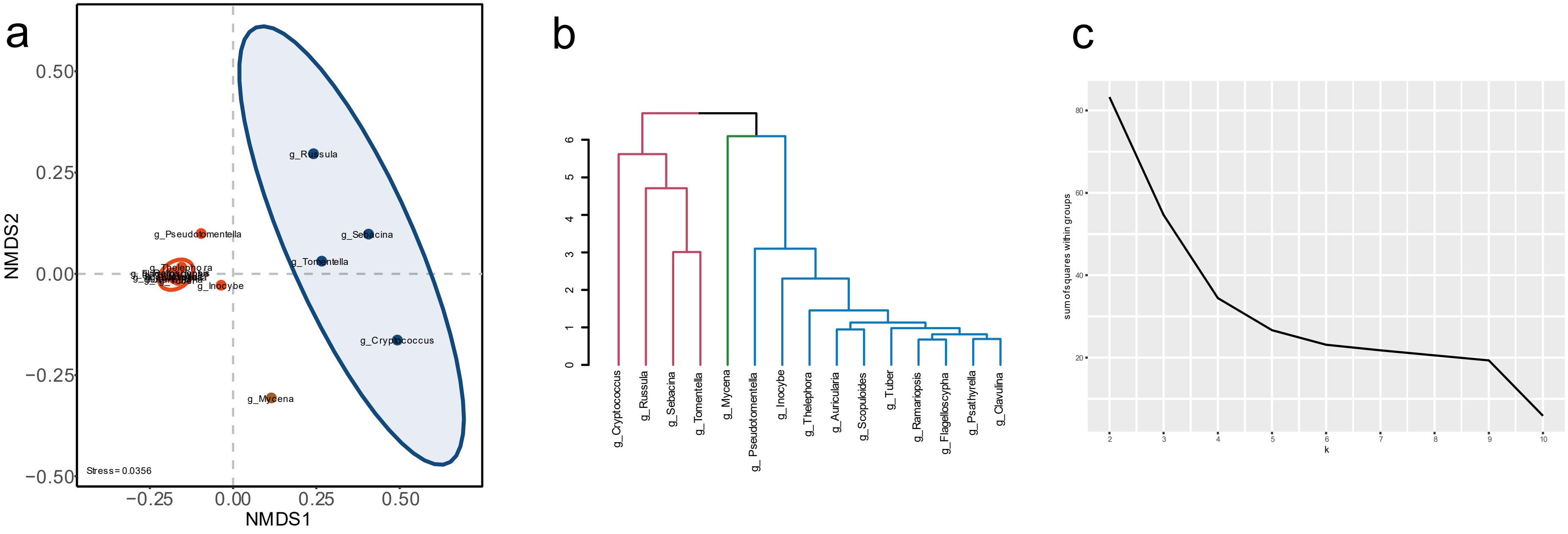

Figure S5 Clustering results of the 15 PGPF Genera.

a: NMDS dimension reduction display, ellipses were plotted according to the 95% confidence interval, distance algorithm were Gower.

b: hierarchical clustering results, method: avrange linkage.

c: elbow plot of k-means clustering
